# Complement alone drives efficacy of a chimeric antigonococcal monoclonal antibody

**DOI:** 10.1371/journal.pbio.3000323

**Published:** 2019-06-19

**Authors:** Sunita Gulati, Frank J. Beurskens, Bart-Jan de Kreuk, Marcel Roza, Bo Zheng, Rosane B. DeOliveira, Jutamas Shaughnessy, Nancy A. Nowak, Ronald P. Taylor, Marina Botto, Xianbao He, Robin R. Ingalls, Trent M. Woodruff, Wen-Chao Song, Janine Schuurman, Peter A. Rice, Sanjay Ram

**Affiliations:** 1 Division of Infectious Diseases and Immunology, University of Massachusetts Medical School, Worcester, Massachusetts, United States of America; 2 Genmab, Utrecht, the Netherlands; 3 University of Virginia, Charlottesville, Virginia, United States of America; 4 Center for Complement and Inflammation Research, Imperial College, London, United Kingdom; 5 Boston University School of Medicine, Boston, Massachusetts, United States of America; 6 School of Biomedical Sciences, The University of Queensland, St. Lucia, Queensland, Brisbane, Australia; 7 Department of Systems Pharmacology and Translational Therapeutics, Perelman School of Medicine, University of Pennsylvania School of Medicine, Philadelphia, Pennsylvania, United States of America; Brigham and Women's Hospital, UNITED STATES

## Abstract

Multidrug-resistant *Neisseria gonorrhoeae* is a global health problem. Monoclonal antibody (mAb) 2C7 recognizes a gonococcal lipooligosaccharide epitope that is expressed by >95% of clinical isolates and hastens gonococcal vaginal clearance in mice. Chimeric mAb 2C7 (human immunoglobulin G1 [IgG1]) with an E430G Fc modification that enhances Fc:Fc interactions and hexamerization following surface-target binding and increases complement activation (HexaBody technology) showed significantly greater C1q engagement and C4 and C3 deposition compared to mAb 2C7 with wild-type Fc. Greater complement activation by 2C7-E430G Fc translated to increased bactericidal activity in vitro and, consequently, enhanced efficacy in mice, compared with “Fc-unmodified” chimeric 2C7. Gonococci bind the complement inhibitors factor H (FH) and C4b-binding protein (C4BP) in a human-specific manner, which dampens antibody (Ab)-mediated complement-dependent killing. The variant 2C7-E430G Fc overcame the barrier posed by these inhibitors in human FH/C4BP transgenic mice, for which a single 1 μg intravenous dose cleared established infection. Chlamydia frequently coexists with and exacerbates gonorrhea; 2C7-E430G Fc also proved effective against gonorrhea in gonorrhea/chlamydia-coinfected mice. Complement activation alone was necessary and sufficient for 2C7 function, evidenced by the fact that (1) “complement-inactive” Fc modifications that engaged Fc gamma receptor (FcγR) rendered 2C7 ineffective, nonetheless; (2) 2C7 was nonfunctional in *C1q*^*−/−*^ mice, when C5 function was blocked, or in *C9*^*−/−*^ mice; and (3) 2C7 remained effective in neutrophil-depleted mice and in mice treated with PMX205, a C5a receptor (C5aR1) inhibitor. We highlight the importance of complement activation for antigonococcal Ab function in the genital tract. Elucidating the correlates of protection against gonorrhea will inform the development of Ab-based gonococcal vaccines and immunotherapeutics.

## Abstract 2

A chimeric antibody that contains a "complement-enhancing" mutation in Fc (so-called HexaBody technology) shows increased bactericidal activity compared to antibody bearing wild-type Fc and may represent a promising immunotherapeutic approach against multidrug-resistant gonorrhea.

## Introduction

Gonorrhea is the second most commonly reported bacterial sexually transmitted infection (STI) worldwide. An estimated 78 million cases of gonorrhea occur globally each year [1]. *N*. *gonorrhoeae*, the causative agent of gonorrhea, has become resistant to almost every conventional antimicrobial currently in clinical use. The emergence of multidrug-resistant isolates of *N*. *gonorrhoeae* worldwide [2] necessitates the need for the urgent development of safe and effective vaccines and novel therapies [3].

Antibodies provide a safe and effective means to target bacteria selectively for complement-mediated killing and/or elimination through opsonophagocytosis. Targeting surface epitopes expressed by most clinical isolates is important to attain broad strain coverage, a critical consideration for *N*. *gonorrhoeae* in which several outer membrane structures, including proteins [4] and lipooligosaccharides (LOSs) [5, 6], are under the control of phase-variable genes. Further, directing the antibody (Ab) toward epitope structures that are critical for fitness of the organism would limit the development of resistance.

Gonococcal LOS is important in several facets of pathogenesis, including cellular adhesion [7], complement resistance [8–10], interactions with immune cells [11, 12], and modulation of inflammation [13]. Despite phase variation, *N*. *gonorrhoeae* LOS, because of its surface density, has the potential to be readily targeted by an immunotherapeutic Ab. Our group identified an epitope on gonococcal LOS that is recognized by a monoclonal Ab (mAb) called 2C7 (and therefore referred to as the “2C7 epitope”), which is expressed on 94% of gonococci (64 out of 68) recovered directly from human cervical secretions [14]. Passive administration of mAb 2C7, as well as active immunization with a peptide mimic (mimitope) of the 2C7 epitope, configured as a “multi-antigen peptide” on a poly-lysine “backbone” [15], shortened the duration and burden of infection significantly in the murine vaginal colonization model of gonorrhea [16]. Taken together, these data suggest that the 2C7 epitope is a promising target both for a therapeutic mAb and as a vaccine.

Upon binding of the F(ab)_2_ regions of immunoglobulin G (IgG) to their cognate epitopes on surfaces, Fc regions form ordered hexamers that can engage the six globular heads of C1q in the C1 complex and initiate activation of the classical pathway [17, 18]. Certain amino acid changes in the Fc region that enhance Fc:Fc interactions can increase IgG hexamer formation, which, in turn, augments complement activation [17, 19]. Engineering of the Fc region in this manner can increase the efficacy of therapeutic antibodies that rely on membrane attack complex (MAC) (C5b-9)-mediated cell lysis (or killing) for their mechanism of action. Here, we examined the effects of a complement-enhancing Fc variant (E430G)—under development to combat multidrug-resistant gonorrhea—on the efficacy of an engineered chimeric mAb 2C7. Further, we define the mechanism of action of this chimeric mAb in vivo.

## Results

### Fc variants do not alter binding of chimeric mAb 2C7 to *N*. *gonorrhoeae*

The epitope in gonococcal LOS recognized by mAb 2C7 and the amino acid variable domain, heavy chain (V_H_) and variable domain, light chain (V_L_) (lambda) sequences of mAb 2C7 used to create the chimeric mAb 2C7 molecules are shown in S1A Fig. The following mAb 2C7–derived chimeric Ab molecules that each contain mouse variable regions (murine V_H_ and V_L_) and human IgG1 heavy- and light-chain constant regions (human C_H_1, C_H_2, and C_H_3 and human C_L_) (S1B Fig) were examined for their ability to bind to *N*. *gonorrhoeae* strain FA1090: (1) chimeric mAb 2C7 with wild-type (WT) human IgG1 Fc; (2) chimeric mAb 2C7 with an E-to-G change at position 430 in human IgG1 Fc at the interface of C_H_2 and C_H_3 that, upon binding to tumor cells, has been demonstrated to have enhanced Fc hexamerization and complement activation; and (3) chimeric mAb 2C7 in which D270 and K322 in C_H_2 were replaced with Ala (A) that resulted in decreased C1q binding [20] (S1B Fig shows a schematic of the chimeric molecule and location of the Fc amino acid changes). As expected, the Fc modifications did not alter binding of the three chimeric mAb 2C7 molecules to FA1090 across concentrations ranging from 1 to 100 μg/mL (S1C Fig).

### Chimeric mAb 2C7-E430G shows increased serum bactericidal activity

**Fig 1:**
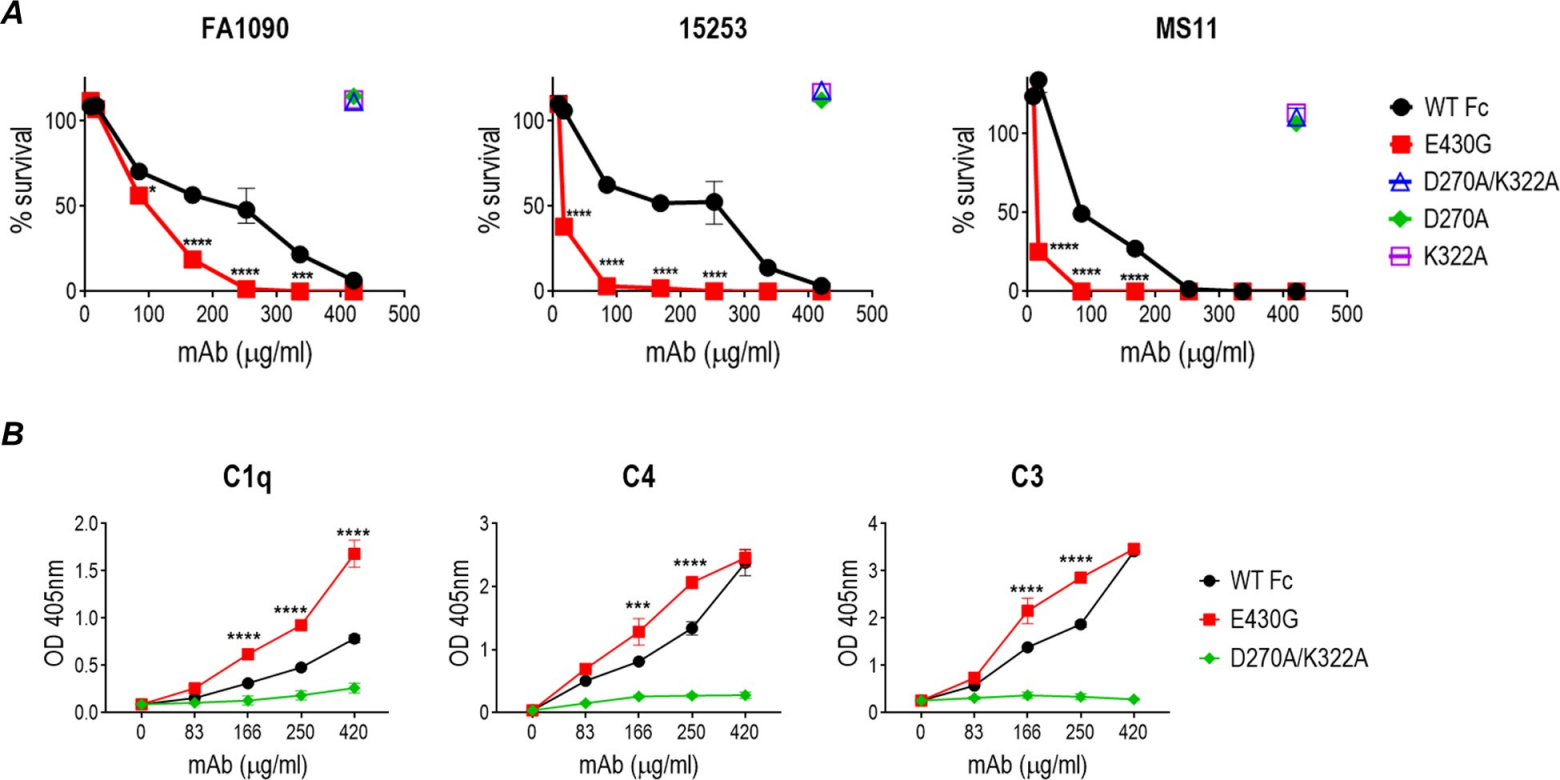
The E430G Fc variant of chimeric mAb 2C7 enhances complement-dependent bactericidal activity and complement deposition on *N*. *gonorrhoeae* compared to WT Fc. (A) Serum bactericidal activity of chimeric mAb 2C7 and its 2C7 Fc variants against *N*. *gonorrhoeae* strains FA1090 (left graph), 15253 (middle graph), MS11 (right graph) in the presence of 16.7% NHS for FA1090 and 15253, or 16.7% human complement (IgG- and IgM-depleted human complement; Pel-Freez) for MS11 as the complement sources. Human complement (IgG/IgM depleted) was used for MS11 because it displays intermediate serum resistance and is only partially resistant to NHS. X-axis, concentration of mAb (μg/mL); y-axis, percent survival (mean [range]) at 30 min relative to counts at 0 min. Two-way ANOVA was used to compare survival across WT Fc and E430G Fc at the various concentrations. *P < 0.05; ***P < 0.001; ****P < 0.0001. (B) The E430G Fc variant enhances C1q binding and C4 and C3 deposition on *N*. *gonorrhoeae*. Strain FA1090 was incubated with mAb 2C7, either with WT Fc, E430G Fc, or D27A/K322A Fc (concentrations indicated on the x-axis) followed by the addition of 16.7% human complement (IgG- and IgM-depleted NHS) for 30 min at 37°C. C1q binding and C4 and C3 deposition on *N*. *gonorrhoeae* were measured by whole-cell ELISA (mean [range] of two experiments). Differences in complement deposition between WT Fc and E430G Fc at the different concentrations were compared by two-way ANOVA. ***P < 0.001; ****P < 0.0001. Data associated with this figure can be found in the supplemental data file (S1 Data). IgG, immunoglobulin G; IgM, immunoglobulin M; mAb, monoclonal antibody; NHS, normal human serum; OD, optical density; WT, wild-type.

Introduction of the E430G change significantly enhanced the bactericidal activity of chimeric mAb 2C7, against three strains of *N*. *gonorrhoeae* (Fig 1A). Whereas strains 15253 and FA1090 are both highly resistant to killing by normal human serum (NHS) [21], strain MS11 displays intermediate complement resistance [22] and therefore was also the most susceptible to complement-dependent killing by mAb 2C7. Carbohydrate extensions beyond lactose substituted heptose I (HepI) of gonococcal LOS can decrease mAb 2C7 binding [23]. As an example, strain 15253 expresses only lactose from HepI and HepII simultaneously (the minimal carbohydrate-substituted LOS structure required for mAb 2C7 binding) [6, 24] and binds more mAb 2C7 than strain FA1090, which expresses lacto-*N*-neotetraose, resulting in less susceptibility to complement-mediated mAb 2C7-E430G–dependent killing than 15253 has. As expected, the molecule that lacked the ability to activate complement, D270A/K322A, did not kill strain FA1090 in the presence of complement. We also created single D270A and K322A variants and tested their ability to mediate complement killing (bactericidal reactivity in NHS). These Fc variants were not expected to engage C1q but differed in their ability to bind Fc gamma receptors (FcγRs), a family of receptors that bind the Fc domain of IgG and facilitate opsonophagocytosis (discussed below). All three tested gonococcal strains were fully resistant to killing (>100% survival) by these “complement-inactive” Fc variants in serum bactericidal assays (Fig 1A).

Whole-cell ELISA examined C1q binding and C4 and C3 deposition on FA1090 in the presence of mAb 2C7 with WT Fc compared with 2C7Ab that possessed E430G Fc (Fig 1B). The E430G Fc variant bound more C1q than the WT Fc. The D270A/K322A variant, used as a negative control, bound a negligible amount of C1q. Likewise, the E430G Fc variant deposited more C4 and C3 on FA1090 than WT Fc did when used at concentrations of 166 and 250 μg/ml, at which differences in serum bactericidal activity were also noted (Fig 1A, left graph). When the concentration of the chimeric mAbs was increased to 420 μg/ml, there was no difference in C4 and C3 deposition mediated by WT Fc and E430G Fc, despite more C1q engaged by bacteria-bound 2C7-E430G Fc, suggesting a limitation in downstream complement activation despite increases in C1q binding.

### Efficacy of mAb 2C7 in vivo correlates with its ability to activate complement

**Fig 2:**
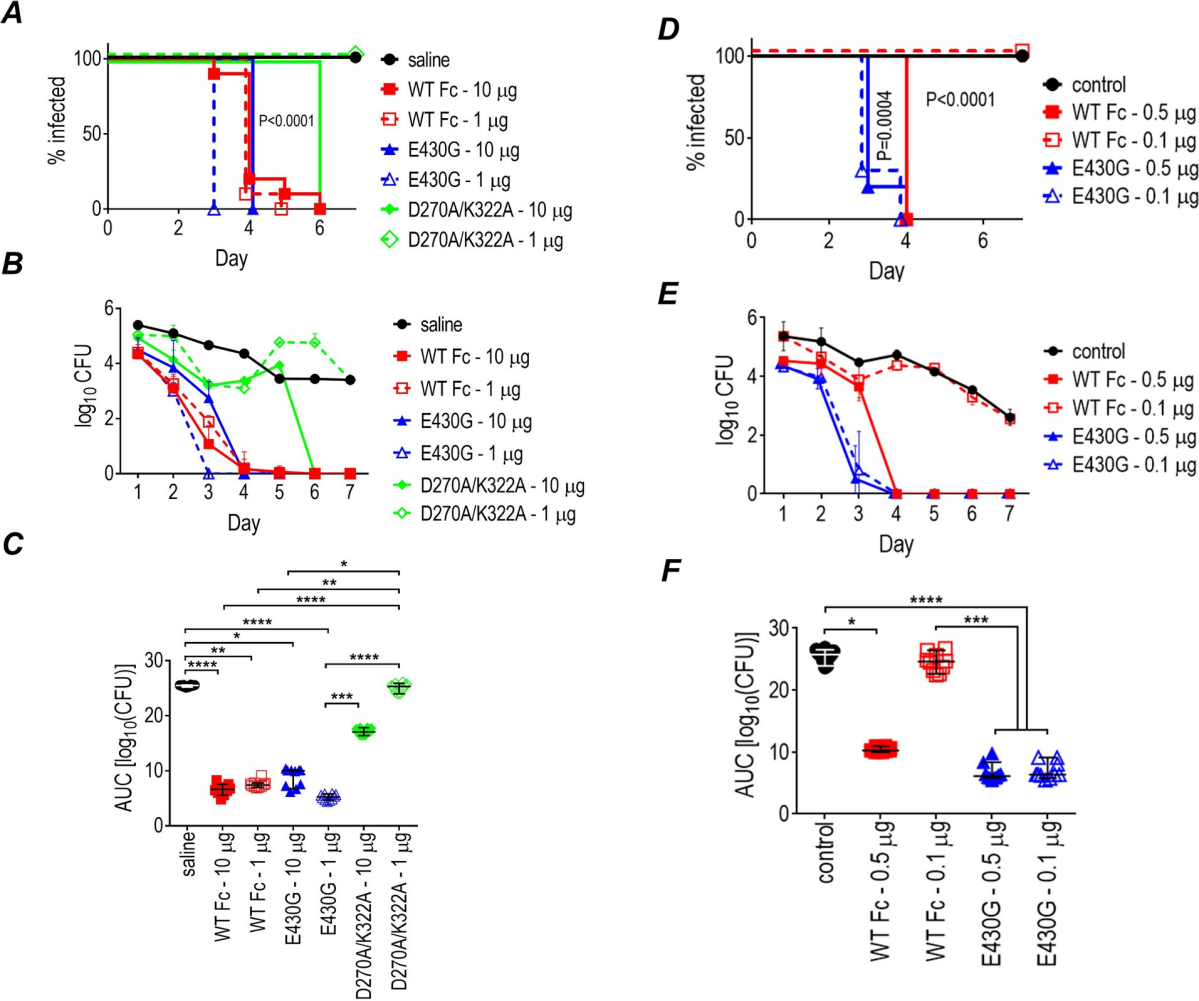
Activity of chimeric mAb 2C7 and 2C7 Fc variant derivatives against *N*. *gonorrhoeae* FA1090 in the mouse vaginal colonization model. (A-C) Mice (*n* = 10/group) were challenged with 8 × 10^5^ CFU FA1090, and mAb 2C7 or its derivatives was administered intravaginally daily at either 10 μg/d or 1 μg/d over the 7 d course of the experiment. The vaginal cavity was swabbed daily and recovered CFU enumerated. (A) Kaplan Meier graph showing time to clearance of infection (*P* < 0.0001 when groups treated with 2C7-WT Fc or 2C7-E430G Fc were compared with saline [control] or D270A/K322A groups; Mantel-Cox analysis). Significance was set at 0.002 (Bonferroni correction for 7 groups). *P* < 0.0001 for mAb 2C7-E430G (1 μg/d) versus 1 μg/d mAb 2C7-WT Fc (1 μg/d). (B) Log_10_ CFU versus time (mean [SD]). (C) AUC analysis to compare bacterial burdens over time. The median and 95% confidence interval are shown for each group. A one-way ANOVA showed significant differences across treatment groups (*P* < 0.0001 by Kruskal-Wallis nonparametric test). Pairwise comparisons across groups was performed using Dunn’s post hoc test. *P < 0.05; **P < 0.01; ***P < 0.001; ****P < 0.0001. (D-F) Activity of lower doses (0.5 and 0.1 μg) given to mice (*n* = 10/group) intravaginally (daily) of chimeric mAb 2C7 and 2C7-E430G Fc. Mice were administered mAb 2C7 or mAb 2C7-E430G intravaginally daily at either 0.5 μg/d or 0.1 μg/d over 7 d and were challenged with 8.75 × 10^6^ CFU FA1090. (D) Kaplan Meier graph showing time to clearance of infection. Significance was set at 0.005 (Bonferroni correction for 5 groups). *P* < 0.0001 for WT Fc (0.5 μg/d) and E430G Fc (0.1 and 0.5 μg/d) versus saline (control) or WT Fc at 0.1 μg/d (Mantel-Cox analysis). WT Fc (1 μg/d) versus E430G Fc (1 μg/d), *P* = 0.0004. (E) Log_10_ CFU versus time (mean [SD]). (F) AUC analysis. The median and 95% confidence interval are shown for each group. Comparison across groups by one-way ANOVA showed significance (*P* < 0.0001). Pairwise comparisons across groups were made using Dunn’s post hoc test. *P < 0.05; ***P < 0.001; ****P < 0.0001. Data associated with this figure can be found in the supplemental data file (S1 Data). AUC, area under curve; CFU, colony-forming units; mAb, monoclonal antibody; WT, wild-type.

We sought to determine whether enhanced complement activation and killing of gonococci in vitro by 2C7-E430G Fc translated to increased activity against *N*. *gonorrhoeae* in the mouse vaginal colonization model. In the first experiment, each of the three mAb 2C7 molecules were administered locally (intravaginally) at doses of either 10 or 1 μg/d (Fig 2A–2C). The two “complement-active” molecules were efficacious at both doses tested when compared with the saline control group. Of note, the median time to clearance (Fig 2A) of the group given 2C7-E430G Fc at 1 μg daily (3 d) was faster than the 2C7-WT Fc group given the same dose (4 d; *P* < 0.0001). A comparison of area under curve (AUC), a measure of the cumulative burden of infection over time (Fig 2C), also showed significantly decreased bacterial burdens in the group that received 1 μg 2C7-E430G daily or 2C7-WT Fc compared to the saline control groups. The complement-inactive D270A/K322A molecule showed minimal activity at the higher (10 μg/d) dose but no activity at 1 μg/d, highlighting the importance of complement activation for efficacy of 2C7.

We next administered lower doses of 0.5 and 0.1 μg/d to attempt to elucidate functional differences between the E430G Fc and the WT Fc molecules (Fig 2D–2F). Although both molecules were effective at 0.5 μg/d, only E430G Fc showed activity at 0.1 μg/d. Similar to the previous observation with the 1 μg/d dose, the median time to clearance in mice given 0.5 μg of 2C7-E430G Fc (3 d) was again faster than the clearance time for mice given the same dose of the WT Fc molecule (4 d; *P* = 0.0004).

The greater efficacy of E430G Fc and inactivity of the “complement-inactive” variant was confirmed in a second separate experiment using intravaginal doses of 1, 0.5, and 0.1 μg of 2C7-WT Fc and 2C7-E430G Fc and 10 μg of the inactive D270A/K322A Fc variant (S2 Fig).

### Activity of mAb 2C7 requires complement activation but not the ability to engage Fcγ receptors

The D270A/K322A variant was constructed to abrogate C1q binding and thereby isolate the role of mAb 2C7’s complement activation ability from its ability to engage FcγR of professional phagocytes, neutrophils in particular [25, 26]. However, previous reports showed that, in comparison with binding by WT human Fc, human IgG1 with a D270A change in the Fc region resulted in >85% reduction in binding to FcγRIIA, FcγRIIB, and FcγRIIIA; this decrease should be compared to the K322A variant, which resulted in only about a 2-fold reduction in binding to FcγRIIIA [27, 28]. Here, we sought to differentiate the roles of complement activation and FcR engagement by creating (1) a D270A Fc (single) variant, which we hypothesized would neither activate complement nor fully bind FcγR, and (2) a K322A Fc (single) variant, which would not activate complement but would bind FcγRs with similar affinity as WT Fc.

**Fig 3:**
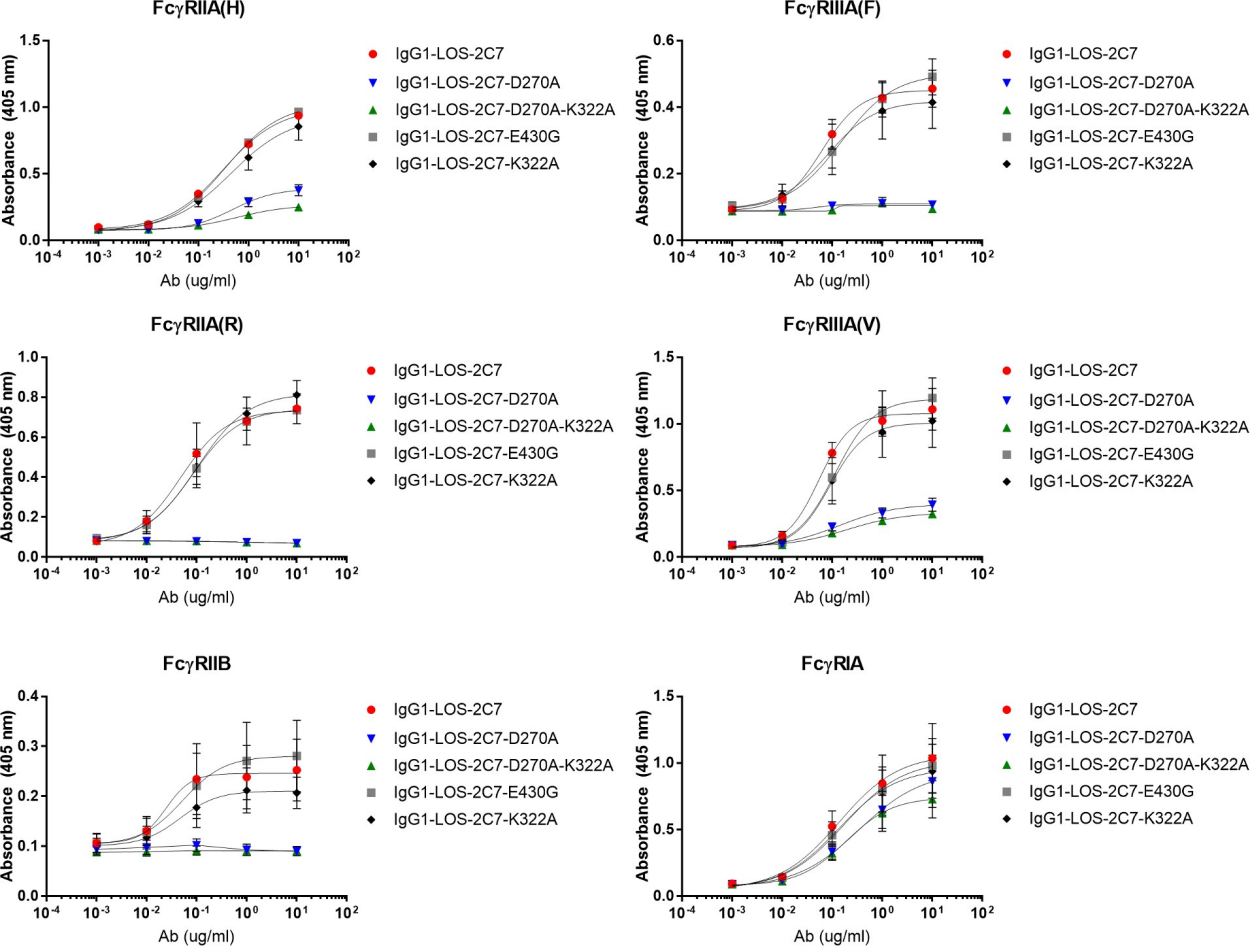
Binding to FcγR of chimeric 2C7 Fc variant mAbs. For binding of mAb 2C7 Fc variants to dimeric variants of ECDs of FcγRIIA allotype 131H, FcγRIIA allotype 131R, FcγRIIB, FcγRIIIA allotype 158F, and FcγRIIIA allotype 158V, increasing concentrations (x-axis) of chimeric 2C7 Fc variant mAbs were dispensed to and incubated with goat F(ab’)_2_-anti-human-IgG-F(ab’)_2_-coated microtiter wells. Subsequent binding of FcγRs is shown as absorbance at 405 nm (y-axis). For binding to monomeric ECD of FcγRIA, increasing concentrations (x-axis) of chimeric 2C7 mAbs with wild-type Fc or Fc variants were dispensed into microtiter wells coated with FcγRIA. Binding is shown as absorbance at 405 nm (y-axis). Data associated with this figure can be found in the supplemental data file (S1 Data). Ab, antibody; ECD, extracellular domain; FcγR, Fc gamma receptor; IgG, immunoglobulin G; LOS, lipooligosaccharide; mAb, monoclonal Ab.

The two variants listed above plus the “double” D270A/K322A variant, the E430G variant, and WT Fc were all tested for binding, by ELISA, to the following FcγRs: FcγRIA, FcγRIIA allotype H, FcγRIIA allotype R, FcγRIIB, FcγRIIIA allotype F, and FcγRIIIA allotype V (Fig 3; summarized in Table 1). AUC analyses are shown in S3 Fig. Anti-human-IgG-F(ab’)2 was used to capture 2C7 Ab variants on ELISA plates. Use of anti-human-Fcγ confirmed uniform capture of all the Fc variant molecules. Except for FcγRIA, all Fc molecules with the D270A change (the single D270A and the double D270A/K322A variants) were severely compromised in their ability to bind to all allotypes of FcγRIIA, FcγRIIB, and FcγRIIIA. The single K322A Fc change showed approximately 30% reduction in binding to FcγRIIB but otherwise bound comparably to WT Fc and other FcγRs. The E430G Fc molecule showed similar binding to all FcγRs compared to the WT Fc.

**Table 1:** Summary of binding and activity of Fc variants of chimeric mAb 2C7.

Fc variant	Binding to FcγR ^A^^,^^C^						Bactericidal activity	Efficacy in mice ^B^
	IA	IIA(H)	IIA(R)	IIB	IIIA(F)	IIIA(V)		
**Wild type**	++	++	++	++	++	++	++	++
**E430G**	++	++	++	++	++	++	+++	+++
**D270A/K322A**	+	−	−	−	−	−	−	−
**D270A**	+	−	−	−	−	−	−	−
**K322A**	++	++	++	+	++	++	−	−

^A^ Allotype indicated in parenthesis.

^B^ Activity at doses ≤ 1 μg/d, intravaginally; ++, similar to wild type (76%–100% of wild type); +, less (25%–75%) than wild type; +++, greater than (>100%) wild type; −, no (<25% of wild type) binding/activity.

^C^ Binding relative to wild-type Fc is derived from AUC analysis in S3 Fig.

Abbreviations: AUC, area under curve; FcγR, Fc gamma receptor; mAb, monoclonal antibody.

**Fig 4:**
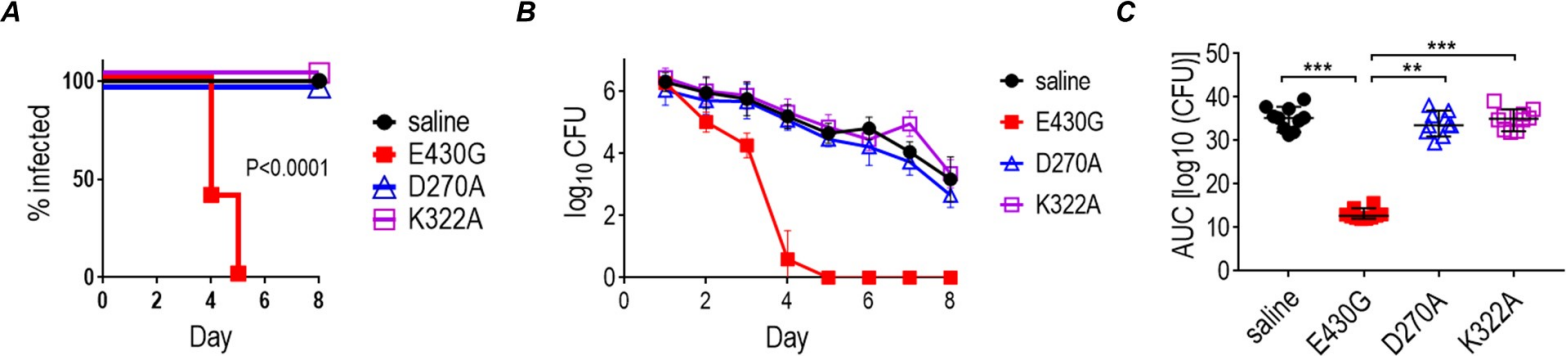
Efficacy of mAb 2C7 requires complement activation by Fc. Wild-type BALB/c mice (*n* = 10/group) were infected with 10^7^ CFU *N*. *gonorrhoeae* FA1090 and treated intravaginally (daily) with 0.5 μg of each of the 2C7 mAb Fc variants. The variant 2C7-E430G Fc served as a positive control for efficacy. (A) Kaplan Meier graph showing time to clearance of infection. Significance was set at 0.008 (Bonferroni correction for 4 groups). *P* < 0.0001 for E430G Fc versus each of the other groups. (B) Log_10_ CFU versus time (mean [SD]). (C) AUC analysis. The median with 95% confidence intervals for each group is shown. Comparison across groups by one-way ANOVA showed significance (*P* < 0.0001 by the Kruskal-Wallis test). Pairwise comparisons across groups using Dunn’s post hoc test. **P < 0.01; ***P < 0.001. Data associated with this figure can be found in the supplemental data file (S1 Data). AUC, area under curve; CFU, colony-forming units; mAb, monoclonal antibody.

The D270A and K322A variants both lacked bactericidal activity (Fig 1A) and therefore provided an opportunity to separate efficacy of mAb 2C7 because of complement activation from FcγR engagement alone. The single D270A and K322A variants were tested for efficacy in mice (Fig 4); the E430G Fc molecule served as a positive control. Each mAb was dosed intravaginally at 0.5 μg/d. The D270A and K322A variants both showed no activity. Thus, complement activation is critical for activity of mAb 2C7, suggested by loss of activity of its K322A Fc derivative, which does not activate complement but binds FcγRs similarly to mAb 2C7 with WT Fc. Results of binding of each mAb 2C7 Fc variant to FcγRs, their bactericidal activities, and efficacy in mice are summarized in Table 1.

### The classical and terminal complement pathways are essential for mAb 2C7 activity

**Fig 5:**
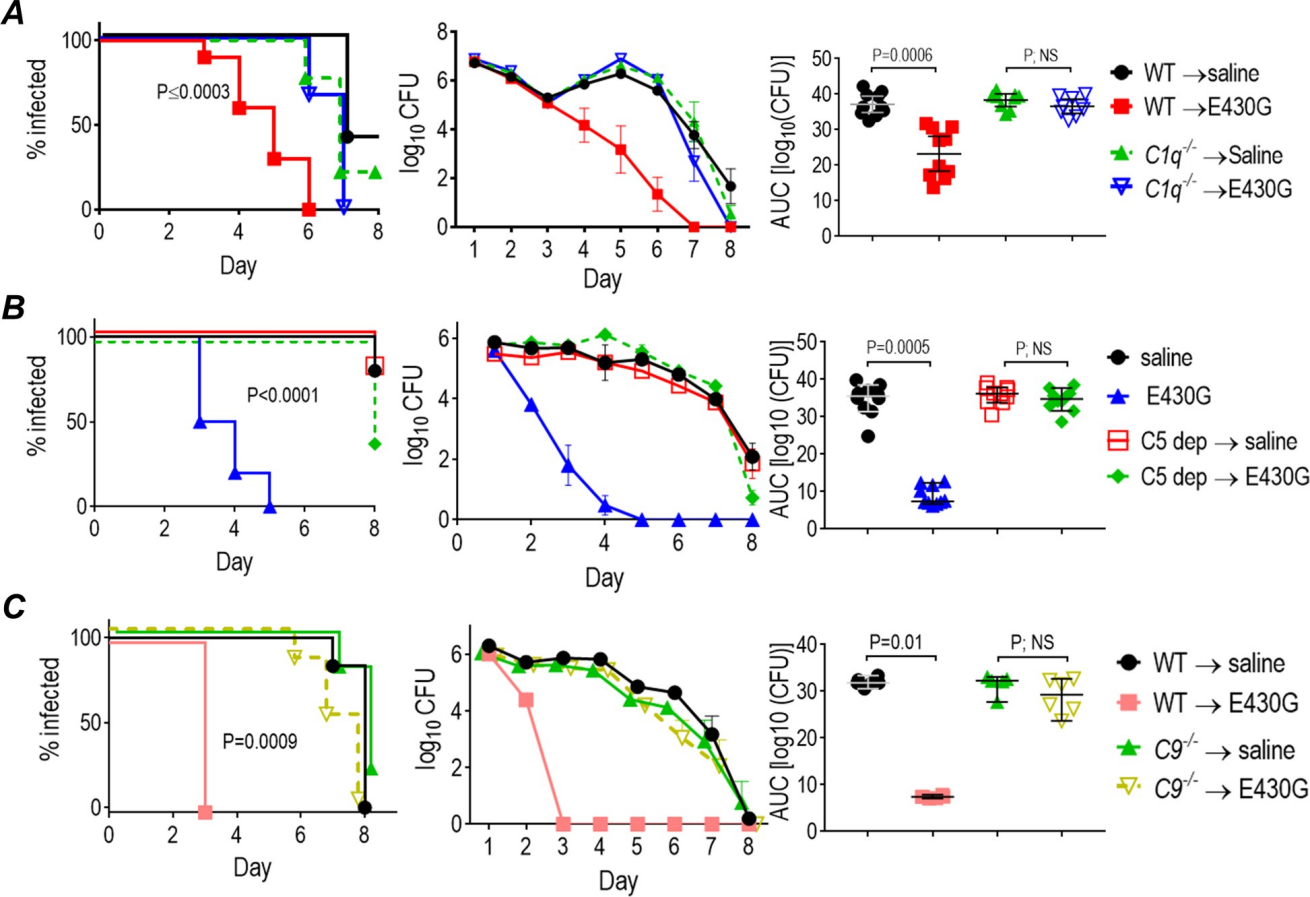
An intact complement system is required for efficacy of mAb 2C7-E430G Fc. (A) mAb 2C7-E430G Fc (abbreviated E430G in the figure) is not efficacious in *C1q*^*−/−*^ mice. *C1q*^*−/−*^ mice and WT C57BL/6 control mice (*n* = 9–10/group) were infected with 4 × 10^6^ CFU *N*. *gonorrhoeae* strain FA1090 and administered saline (control) or intravaginal (daily) 0.1 μg/d of mAb 2C7-E430G Fc. Vaginas were swabbed daily to enumerate CFUs. Left graph: Kaplan Meier graph showing time to clearance of infection (*P* ≤ 0.0003 for E430G-treated WT mice versus all other groups; significance was set at 0.008 [Bonferroni correction for 4 groups]). Middle graph: Log_10_ CFU versus time (mean [SEM]). Right graph: AUC analysis. The median and 95% confidence intervals are shown for each group. Comparison across groups by one-way ANOVA were significant (*P* < 0.0001; Kruskal-Wallis test). Pairwise comparisons across groups were made with Dunn’s post hoc test. (B) C5 blockade function abrogates efficacy of mAb 2C7-E430G Fc. C5 function in WT BALB/c mice was blocked with mAb BB5.1 (1 mg intraperitoneally on days −1, 2, and 5). In addition, 10 μg of mAb BB5.1 was also administered intravaginally (daily for 8 d). Saline was used as a control in C5 sufficient mice. Four groups of mice (*n* = 10/group) infected with *N*. *gonorrhoeae* FA1090 were treated as follows: (1) WT mice, saline intravaginally; (2) C5 blockaded mice, saline intravaginally; (3) WT mice, mAb 2C7-E430G Fc, 0.1 μg intravaginally, daily; and (4) C5 blockaded mice, mAb 2C7-E430G Fc, 0.1 μg intravaginally, daily. Left graph: Kaplan Meier graph showing time to clearance of infection. Significance was set at 0.008 (Bonferroni correction for 4 groups). *P* < 0.0001 for E430G-treated WT mice versus all other groups. Middle graph: Log_10_ CFU versus time (mean [SEM]). Right graph: AUC analysis. The median and 95% confidence intervals are shown for each group. Comparison across groups by one-way ANOVA by Kruskal-Wallis test showed significance (*P* < 0.0001). Pairwise comparisons across groups were made with Dunn’s post hoc test. (C) mAb 2C7-E430G Fc loses efficacy in *C9*^*−/−*^ mice. Four groups of mice (*n* = 5–6/group) were infected with *N*. *gonorrhoeae* FA1090 (3.6 × 10^7^) and were treated as follows: (1) WT mice, saline intravenously; (2) WT mice, mAb 2C7-E430G intravenously (5 μg intravenously, single dose on day +1); (3) *C9*^*−/−*^ mice, saline intravenously; and (4) *C9*^*−/−*^ mice, mAb 2C7 E430G intravenously (5 μg intravenously, single dose on day +1). Left graph: Kaplan Meier graph showing time to clearance of infection. Significance was set at 0.008 (Bonferroni correction for 4 groups). *P* = 0.0009 for E430G-treated WT mice versus all other groups. Middle graph: Log_10_ CFU versus time (mean [SEM]). Right graph: AUC analysis. The median and 95% confidence intervals are shown for each group. Comparison across groups by one-way ANOVA by Kruskal-Wallis test showed significance (*P* = 0.0028). Pairwise comparisons across groups were made with Dunn’s post hoc test. Data associated with this figure can be found in the supplemental data file (S1 Data). AUC, area under curve; CFU, colony-forming units; mAb, monoclonal antibody; NS, not significant; WT, wild type.

Having shown that modifying Fc while sustaining complement activation maintained efficacy of mAb 2C7, we next used mice deficient in individual complement components to assess the roles of the classical and terminal pathways in enabling 2C7 to clear *N*. *gonorrhoeae*. Binding of Ab to surfaces results in formation of ordered Fc hexamers, which then engage C1q. We used *C1q*^*−/−*^ mice to demonstrate a requirement for the classical pathway. Although 2C7-E430G Fc was efficacious in WT C57BL/6 mice, 2C7-E430G Fc lost efficacy in *C1q*^*−/−*^ mice (Fig 5A).

Engagement of FcγR alone (in the absence of complement activation) does not support activity of mAb 2C7. However, simultaneous engagement of complement receptors and FcγR by C3 fragments (mainly iC3b) and IgG Fc, respectively, could clear gonococci through opsonophagocytic killing [29, 30]. If this were the case, selectively blocking the terminal complement pathway, leaving C3 deposition unaffected, would not affect efficacy of mAb 2C7. The first step in assembly of the MAC is cleavage of C5 to C5a, which is released into solution, and C5b, which binds C6 and subsequently forms the C5b-9 complex (MAC) [31]. C5 cleavage and function was blocked using mAb BB5.1. As shown in Fig 5B, mAb 2C7-E430G administered intravaginally was ineffective in mAb BB5.1–treated mice; times to clearance, log_10_ colony-forming units (CFU) versus time, and AUC were similar across the two saline-treated groups and the group of mAb BB5.1–treated animals that received E430G Fc. WT mice that were treated with E430G Fc cleared infection significantly faster than all other groups. This was repeated with a mouse IgG1 isotype control for mAb BB5.1 (S4 Fig) with similar results.

The addition of 12 or more C9 molecules to C5b-8 forms the C5b-9 pore [32–34] and is the final stage in MAC assembly. As shown in Fig 5C, mAb 2C7-E430G Fc lost efficacy in *C9*^*−/−*^ mice. These data show that activity of mAb 2C7 requires intact classical and terminal complement pathways.

### Neutrophils are not required for mAb 2C7 activity

**Fig 6:**
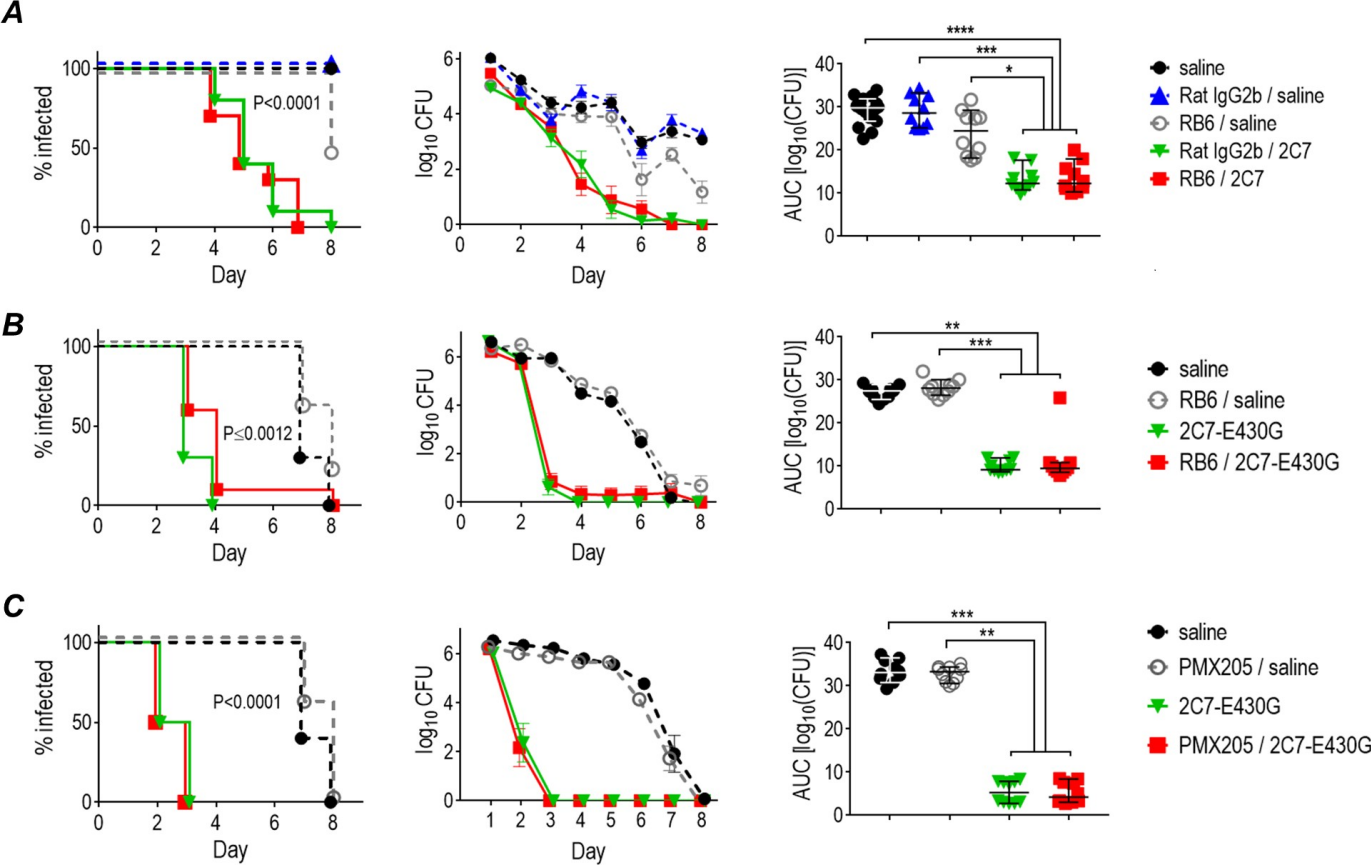
mAb 2C7 activity in the mouse vaginal colonization model does not require PMNs or C5aR1. (A) Efficacy of murine mAb 2C7 (mouse IgG3) in the absence of PMNs. Five groups of BALB/c mice (*n* = 10/group) infected with 10^6^ CFU *N*. *gonorrhoeae* FA1090 were treated as follows: (1) saline (control), (2) RB6 mAb (depletes PMNs), administered IP, (3) RB6 IP plus mAb 2C7 intravaginally, (4) rat IgG2b (control for RB6) IP, and (5) rat IgG2b IP plus mAb 2C7 intravaginally. Left graph: Kaplan Meier curves showing time to clearance of infection. Significance was set at 0.005 (Bonferroni correction for 5 groups). *P* < 0.0001 (Mantel-Cox log-rank test) for each of the groups that received mAb 2C7 versus each of the groups that did not get mAb 2C7. Middle graph: log_10_ CFU versus time (mean [SEM]). Right graph: AUC analysis. The median and 95% confidence interval are indicated for each group. Comparison across groups by one-way ANOVA showed significance (*P* < 0.0001). Groups that received mAb 2C7 showed significantly lower AUCs than each group that did not get mAb 2C7 when compared in a pairwise manner (Dunn’s post hoc test). *P < 0.05; ***P < 0.001; ****P < 0.0001. (B) Chimeric mAb 2C7-E430G maintains efficacy in the absence of PMNs. Four groups of BALB/c mice (*n* = 10/group) infected with *N*. *gonorrhoeae* FA1090 (3.5 × 10^7^ CFU) were treated as follows: (1) saline (control), (2) RB6 mAb, IP, (3) 2C7-E430G (5 μg intravenously on day 1), and (4) RB6 IP and 2C7-E430G intravenously. Left graph: Kaplan Meier curves. Significance was set at 0.008 (Bonferroni correction for 4 groups). *P* = 0.0012 for the two groups that received 2C7-E430G versus each of the groups that did not get 2C7-E430G. Middle graph: log_10_ CFU versus time (mean [SEM]). Right graph: AUC analysis. The median and 95% confidence intervals for each group are shown. Comparison across groups by one-way ANOVA (Kruskal-Wallis test) showed significance (*P* < 0.0001). Pairwise comparisons were made by Dunn’s post hoc test. **P < 0.01; ***P < 0.001. (C) Chimeric mAb 2C7-E430G is effective when C5aR1 is blocked. Mice that had been administered PMX205 (C5aR1 inhibitor) subcutaneously and intravaginally were used in the following experiment. Four groups of BALB/c mice (*n* = 10/group) infected with *N*. *gonorrhoeae* FA1090 (3.2 × 10^7^ CFU) were treated as follows: (1) saline (control), (2) PMX205 (C5aR1 inhibitor), (3) 2C7-E430G (5 μg intravenously on day 1), and (4) PMX205 (C5aR1 inhibitor) and 2C7-E430G. Left graph: Kaplan Meier curves. Significance was set at 0.008 (Bonferroni correction for 4 groups). *P* < 0.0001 for each of the groups that received 2C7-E430G versus each of the groups that did not get 2C7-E430G (Mantel-Cox log-rank test). Middle graph: log_10_ CFU versus time (mean [SEM]). Right graph: AUC analysis. The median and 95% confidence interval are shown for each group. Comparison across groups by one-way ANOVA showed significance (*P* < 0.0001). Pairwise comparisons were made by Dunns’s post hoc test. **P < 0.01; ***P < 0.001. Data associated with this figure can be found in the supplemental data file (S1 Data). AUC, area under curve; C5aR1, C5a receptor; CFU, colony-forming units; IgG, immunoglobulin G; IP, intraperitoneally; mAb, monoclonal antibody; PMN, polymorphonuclear leukocyte.

Freshly isolated human neutrophils and macrophages can kill opsonized gonococci in vitro [35–42]. The data thus far point to a role for complement activation, but not Fc–FcγR interactions, for mAb 2C7 efficacy. To further validate these results, we depleted polymorphonuclear neutrophils (PMNs), the predominant phagocyte present in genital secretions in gonorrhea-infected humans and mice, with mAb RB6. Rat IgG2b was used as the isotype control. The highly serum-bactericidal murine IgG3 mAb 2C7 [16, 43] was used in these experiments to maintain species congruity between Ab Fc and FcγR on PMNs. We used the intraperitoneal route of administration, previously proven to be effective for murine mAb 2C7 [16]. As shown in Fig 6A, murine mAb 2C7 remained active despite depletion of PMNs; PMN depletion was confirmed by complete peripheral blood counts performed on day 5 and also by the lack of PMNs on vaginal smears through day 6. Enumeration of PMNs in the vaginal mucosa as a percentage of the total number of cells is shown in S5 Fig. The observed PMN influx was similar to that reported by Jerse and colleagues in BALB/c mice [44]. Similarly, chimeric mAb 2C7-E430G also remained efficacious when administered as a single intravenous dose of 5 μg given on day 1 in PMN-depleted mice (Fig 6B).

Blockade of C5 with mAb BB5.1 (Fig 5D–5F) prevents C5a generation. C5a is a an important neutrophil chemotaxin for *N*. *gonorrhoeae* [45]. Further, C5a–C5a receptor (C5aR1) interactions are critical for phagocytosis and killing of the related pathogen, *N*. *meningitidis* [46]. Blockade of C5aR1 with PMX205 did not alter the efficacy of mAb 2C7 E430G. Considered together, preservation of mAb 2C7-E430G Fc activity when neutrophils are depleted or when C5aR1 is blocked, but loss of activity in the absence of C9 (Fig 5C), strongly suggests that complement alone is necessary and sufficient for its activity.

### mAb 2C7 is efficacious in transgenic mice that express human factor H (FH) and C4b-binding protein (C4BP)

The ability of *N*. *gonorrhoeae* to evade complement-mediated killing is restricted to humans, in part because they selectively bind to human complement inhibitors such as FH (alternative pathway inhibitor) and C4BP (classical pathway inhibitor). Surface-bound FH and C4BP decrease the bactericidal efficacy of antibodies, including 2C7 [14, 43]. Therefore, we used transgenic (Tg) mice that express human FH and human C4BP (FH/C4BP dual Tg mice) [47] to determine the efficacy of the 2C7 chimeric mAbs to simulate conditions in humans in which mAb 2C7 will need to surmount the effects of these complement inhibitors [48, 49].

**Fig 7:**
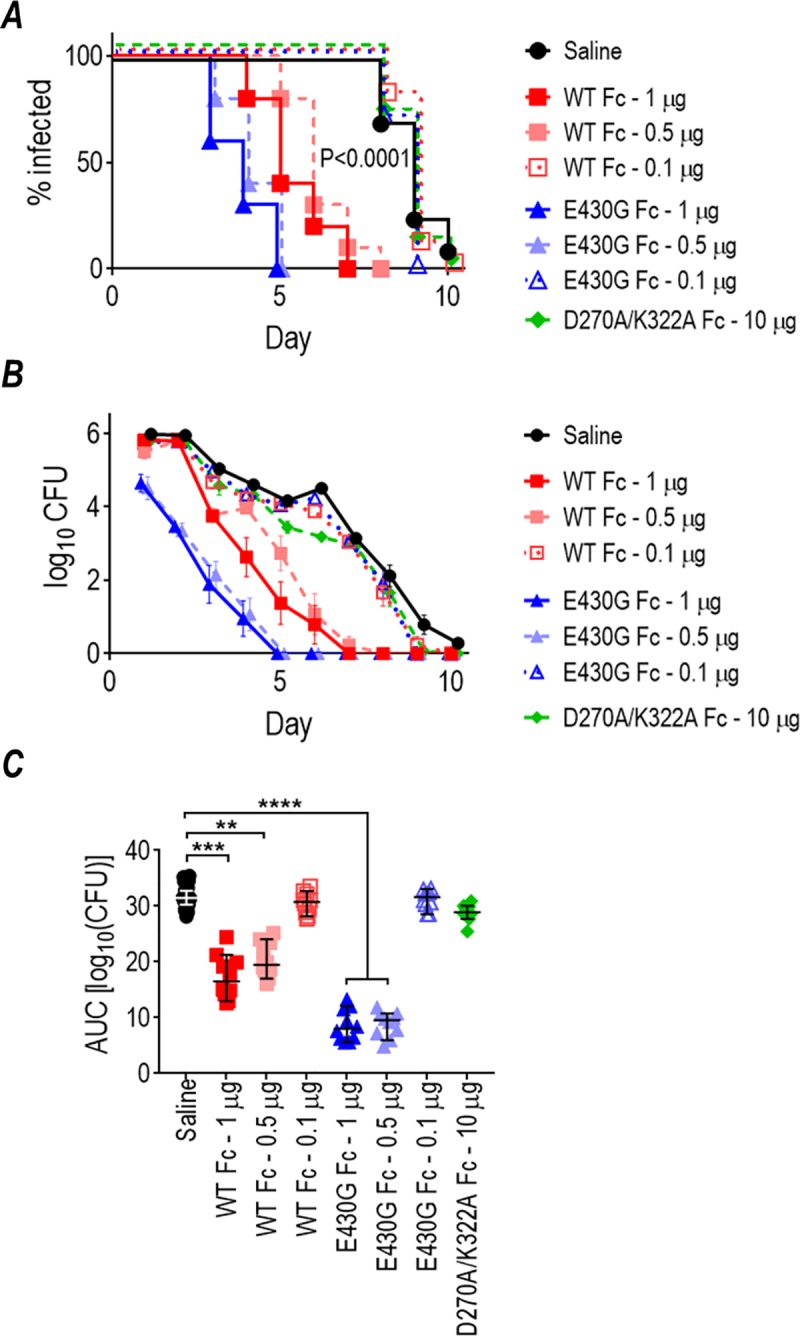
Efficacy of chimeric mAb 2C7 and its Fc variants against *N*. *gonorrhoeae* in human FH/C4BP dual Tg mice. FH/C4BP Tg mice were infected with 8.75 × 10^5^ CFU *N*. *gonorrhoeae* FA1090, which were administered (daily, for 10 d) intravaginally: saline; chimeric WT mAb 2C7 (abbreviated WT Fc) or mAb 2C7-E430G Fc (abbreviated E430G Fc), each of these mAbs at dosages of 1, 0.5, or 0.1 μg/d; or the “complement inactive” 2C7 mAb D270A/K322A Fc (abbreviated D270A/K322A Fc) at 10 μg/d. Vaginal bacterial CFU burdens were enumerated daily. (A) Kaplan Meier graph showing time to clearance of infection. Significance was set at 0.0018 (Bonferroni correction for 8 groups). Groups that received either 1 or 0.5 μg of (2C7) WT Fc cleared infection significantly faster than saline controls, WT Fc and E430G Fc given at the lowest dose (0.1 μg/d), or D270A/K322A Fc. Groups that received 1 or 0.5 μg/d of E430G Fc cleared infection faster than groups that received the corresponding doses of WT Fc (*P* < 0.0001 in all instances). (B) Bacterial burdens (expressed as log_10_ CFU) over time (mean [SEM]). (C) AUC analysis. The median and 95% confidence interval are shown for each group. Comparison across groups by one-way ANOVA were significant (*P* < 0.0001; Kruskal-Wallis test). Pairwise comparisons across groups made using Dunn’s post hoc test. **P < 0.01; ***P < 0.001; ****P < 0.0001. Data associated with this figure can be found in the supplemental data file (S1 Data). AUC, area under curve; C4BP, C4b-binding protein; CFU, colony-forming units; FH, factor H; Tg, transgenic; WT, wild-type.

The presence of human FH and C4BP in the Tg mice resulted in a small but statistically significant enhancement of gonococcal colonization in untreated mice (S6 Fig). Three doses of chimeric 2C7 mAbs with WT Fc or E430G Fc (1, 0.5, and 0.1 μg) or a 10 μg dose of the D270A/K322A Fc, each given intravaginally daily, were tested in dual Tg mice infected with strain FA1090. As shown in Fig 7, only the 1 and 0.5 μg/d doses of the WT Fc and E430G Fc mAbs were efficacious, whereas both mAbs were ineffective at 0.1 μg/d. The lack of efficacy of the 0.1 μg/d dose of the E430G Fc variant in Tg mice contrasts with its efficacy in WT mice (Fig 2). The variant 2C7-E430G at 0.5 μg/d cleared the infection significantly faster than observed for 0.5 μg/d of the WT Fc molecule (Fig 7A; median time to clearance 4 versus 6 d; *P* = 0.0001) and showed a more rapid rate of decline in CFU versus time (Fig 5B). Treatment with 2C7-E430G Fc at 1 and 0.5 μg/d resulted in approximately a 2-fold lower AUC than animals treated with the corresponding doses of 2C7-WT Fc (*P* < 0.0001 in both instances).

The efficacy of intravaginally administered WT Fc and E430G Fc at dosages of 1 and 0.5 μg/d in the dual Tg mice was evaluated using two additional *N*. *gonorrhoeae* strains, MS11 (expresses Porin B [PorB.1B]; S7A–S7C Fig) and 15253 (expresses PorB.1A; S7D–S7F Fig). Both mAbs significantly reduced the median time to clearance of both strains at each dose. The variant 2C7-E430G Fc significantly reduced the burden of MS11 infection (measured as AUC) compared to the corresponding dose of WT Fc (S7C Fig). Strain 15253 was less virulent compared to MS11; in animals treated with saline only, 15253 infection had cleared by 9 d, whereas 50% of animals infected with MS11 were still infected at 9 d (*P* = 0.0004), and 15253-infected animals showed lower consolidated bacterial burdens over time (AUC analysis; *P* = 0.0005). Both mAbs were similarly efficacious in clearing infection with 15253 (S7D–S7F Fig). In conclusion, 2C7-E430G Fc is also effective in human FH/C4BP Tg mice and shows greater efficacy than 2C7-WT Fc.

### Efficacy of systemically administered mAb 2C7 E430G for colonization

**Fig 8:**
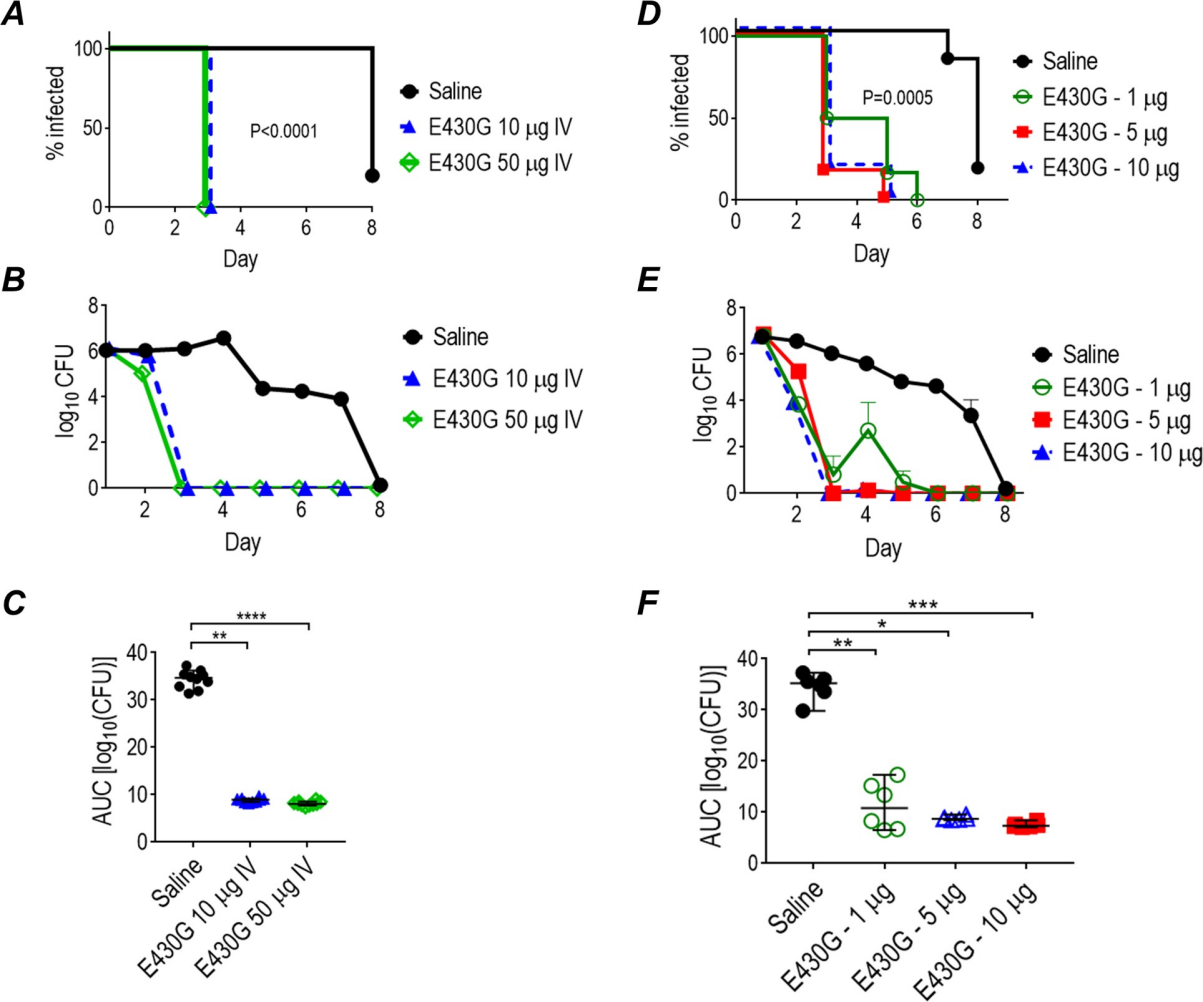
Systemically administered mAb 2C7-E430G Fc (abbreviated E430G) administered at either high (10 μg or 50 μg) or low (1 μg, 5 μg, and 10 μg [the latter as overlapping]) doses clears vaginal gonococcal infection in human FH/C4BP transgenic mice. (A-C) Human FH/C4BP female transgenic mice (*n* = 10/group) were infected intravaginally with 6 × 10^7^ CFU *N*. *gonorrhoeae* FA1090 and treated 24 h later with a single dose of 2C7-E430G (either 10 μg or 50 μg) or with saline (vehicle control). Vaginas were swabbed daily to enumerate gonococcal CFU. (A) Kaplan Meier curves showing time to clearance. Significance was set at 0.017 (Bonferroni correction for 3 groups). *P* < 0.0001 for each treatment group versus the saline control group. (B) Log_10_ CFU versus time (mean [SEM]). (C) AUC analysis. The median and 95% confidence interval are shown for each group. Comparison across groups by one-way ANOVA were significant (*P* < 0.0001 by the Kruskal-Wallis test). Pairwise comparisons across groups were made using Dunn’s nonparametric test. **P < 0.01; ****P < 0.0001. (D-F) Efficacy of a single 1 μg, 5 μg, and 10 μg IV dose of 2C7-E430G Fc against *N*. *gonorrhoeae* in dual FH/C4BP transgenic mice. Four groups of FH/C4BP transgenic mice (*n* = 6/group) were infected intravaginally with 6.6 × 10^7^ CFU *N*. *gonorrhoeae* FA1090 and treated 24 h later with a single IV injection of 2C7-E430G Fc at the indicated doses. The control group received saline. (D) Kaplan Meier curves showing time to clearance. Significance was set at 0.008 (Bonferroni correction for 4 groups). *P* < 0.0001 for each treatment group versus the saline control group. (E) Log_10_ CFU versus time (mean [SEM]). (F) AUC analysis. Comparisons across groups made using one-way ANOVA were significant (*P* = 0.0012; Kruskal-Wallis test). Pairwise comparisons across groups were made using Dunn’s nonparametric test. Data associated with this figure can be found in the supplemental data file (S1 Data). AUC, area under curve; C4BP, C4b-binding protein; CFU, colony-forming units; FH, factor H; IV, intravenous; WT, wild-type.

mAb 2C7 could be used systemically ad hoc as an immunoprophylactic to reduce the likelihood of acquiring gonorrhea or to attenuate/reduce infection and probably reduce the likelihood of transmission to sex partners [50]. We administered 2C7-E430G Fc systemically (IV) as a single dose to treat colonized mice, as might be used in clinical practice. In the first experiment, FH/C4BP Tg mice were administered either 50 μg or 10 μg intravenously (IV) as a single dose 24 h after intravaginal challenge with FA1090. As shown in Fig 8A–8C, both doses significantly shortened the duration of infection and bacterial burdens over time. A second experiment tested intravenous doses of 10, 5, and 1 μg (Fig 8D–8F). Even the lowest dose used (1 μg) proved effective in attenuating vaginal infection.

### mAb 2C7-E430G is effective in clearing gonorrhea in gonorrhea/chlamydia-coinfected mice

Coinfection with chlamydia and gonorrhea is a common clinical occurrence. In some series, up to 40% of individuals with gonorrhea are coinfected with chlamydia [51, 52]. Chlamydia coinfection is associated with higher gonococcal loads in women [53] and decreased the antigonococcal activity of a meningococcal vaccine that afforded some cross protection against gonorrhea in a retrospective study [54]. Demonstrating efficacy of mAb 2C7 in the context of chlamydia infection is therefore important.

**Fig 9:**
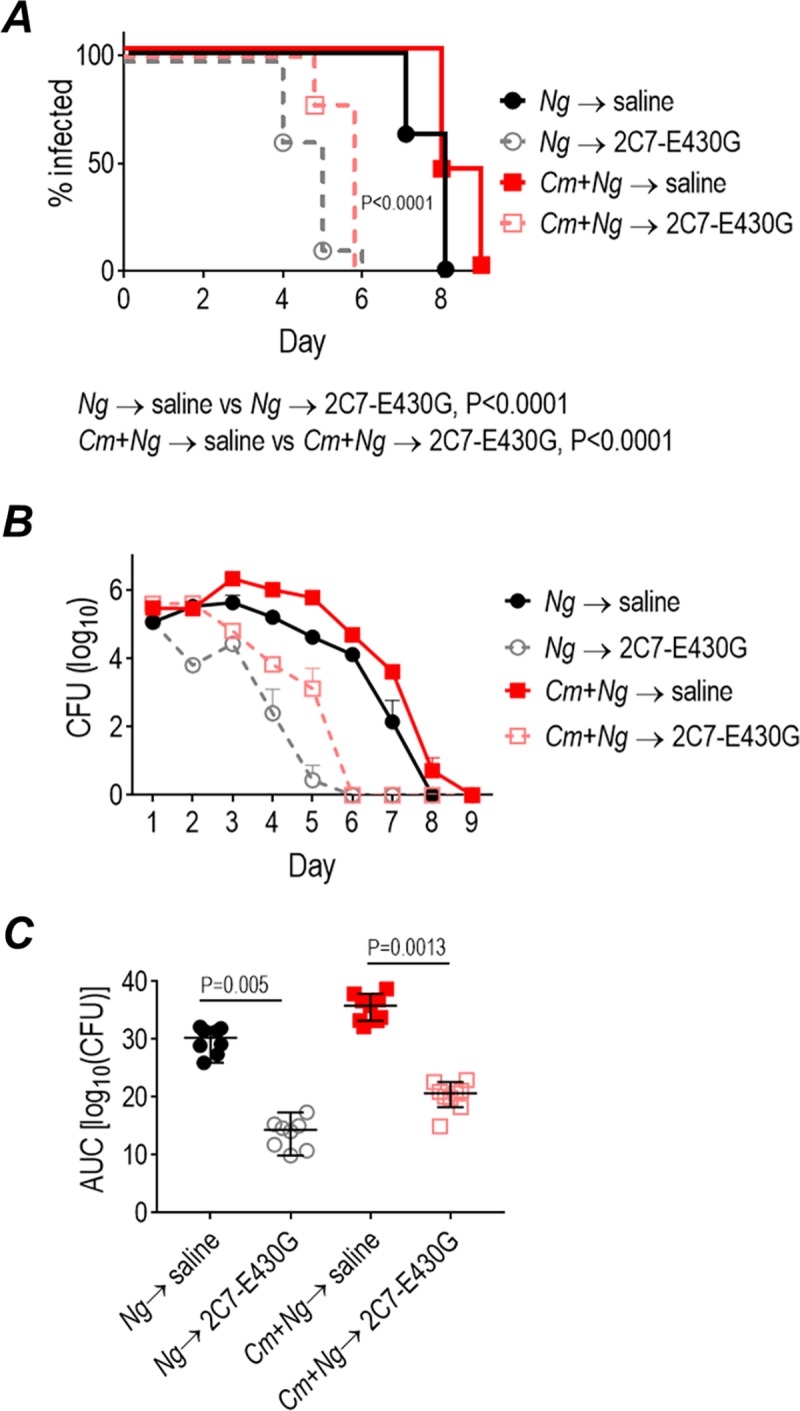
mAb 2C7-E430G Fc (abbreviated 2C7-E430G) is efficacious against *N*. *gonorrhoeae* in FH/C4BP transgenic mice coinfected with *C*. *muridarum*. Human FH/C4BP transgenic mice were infected intravaginally with 2.5 × 10^6^ IFU *C*. *muridarum* on days −4, −3, and −2, followed by infection with 7 x 10^7^ CFU *N*. *gonorrhoeae* FA1090 on day 0 (“*Cm+Ng*”; *n* = 9/group). Mice infected with *N*. *gonorrhoeae* only (“*Ng*”; *n* = 8/group) were used as controls. Mice were treated with 2C7-E430G Fc (1 μg intravaginally, daily for 9 d) or with saline (vehicle controls). (A) Kaplan Meier curves show time to clearance. Significance was set at 0.008 (Bonferroni correction for 4 groups). *P* < 0.0001 for each treatment group versus the corresponding saline control group (*P* < 0.0001). (B) Log_10_ CFU versus time (mean [SEM]). (C) AUC analysis. The median and 95% confidence interval are shown for each group. Comparison across groups made by one-way ANOVA was significant (*P* < 0.0001; Kruskal-Wallis test). Pairwise comparisons across groups were made with Dunn’s nonparametric post hoc test. Data associated with this figure can be found in the supplemental data file (S1 Data). AUC, area under curve; C4BP, C4b-binding protein; CFU, colony-forming units; FH, factor H; IFU, infectious units.

*Chlamydia muridarum* (a mouse-adapted chlamydia strain) coinfection enhances the burden of gonorrhea in mice [55]. To ensure activity of mAb 2C7 in the context of chlamydia coinfection, we administered mAb 2C7-E430G Fc to FH/C4BP Tg mice coinfected with FA1090 and *C*. *muridarum*. As shown in Fig 9, 2C7-E430G Fc given intravaginally at a dose of 1 μg daily significantly reduced the duration and burden of gonorrhea in coinfected mice.

## Discussion

Here, we have developed a chimeric mAb directed against a *N*. *gonorrhoeae* LOS epitope [14] that is a gonococcal vaccine candidate [15, 16]. The parent murine mAb, called 2C7, showed efficacy against *N*. *gonorrhoeae* in the mouse vaginal colonization model when administered intraperitoneally [16]. Given the global threat of multidrug-resistant gonorrhea [2, 3], an Ab such as mAb 2C7 that recognizes a wide spectrum of gonococcal isolates would be invaluable as an adjunctive immunotherapeutic. We recently showed that lactose from HepII on gonococcal LOS, which is essential for mAb 2C7 binding, can be sialylated [56]. Sialylation of HepII lactose contributes to complement resistance and enhances the ability of gonococci to colonize the mouse genital tract [56]. These data explain the widespread expression of the 2C7 epitope and the reason for decreased virulence of gonococcal mutants that lack HepII lactose [56]. Lactose extensions from HepII require expression of LOS glycosyltransferase G [57], which is under control of the phase-variable LOS glycosyltransferase G *(lgtG)* gene. Thus, resistance to mAb 2C7—if it were to occur because *lgtG* is phase-varied “off” due to slipped-strand mispairing of polyC/G tracts—would result in substantial decrease in bacterial fitness in vivo.

The role of complement in host defense against neisserial infections is well established [58, 59]. Individuals with inherited deficiencies in components of the alternative pathway (e.g., factor D and properdin) or the terminal pathway (C5 through C9) or with acquired defects of the terminal pathway (e.g., C5 inhibition with eculizumab) are highly predisposed to recurrent, invasive meningococcal infections. Recurrent disseminated gonococcal infection (DGI) has also been reported in persons with terminal complement deficiencies [60–62] and C2 deficiency [63]. Ellison and colleagues observed that three of 22 individuals with DGI also had CH50 levels greater than two standard deviations below the mean [64]; one had a complete deficiency of C1r; another had preexisting systemic lupus erythematosus with low C4 levels, and a third had a C8 concentration <40% of normal. In contrast to DGI, which is relatively rare, acquisition of gonorrhea that results in local infection is common—infection rates in women exposed to men infected with gonorrhea are as high as 73% following a single sexual encounter [65]. The high infectivity rates of gonorrhea coupled with the rarity of complement defects make it difficult to ascertain whether complement deficiency increases the risk of uncomplicated gonococcal infection.

Our studies show that an intact complement system is necessary and sufficient for activity of mAb 2C7. Several lines of evidence support this conclusion. First, mAb 2C7 function correlated with its ability to activate complement; the three mAb 2C7 Fc derivatives (D270A, K322A, and D270A/K322A) that were unable to activate complement, and were therefore nonbactericidal in vitro, were also ineffective in clearing infection in vivo. Delayed clearance (at or beyond day 6) was seen when the D270A/K322A variant was used topically at a high dose (10 μg daily), suggesting complement-independent mechanism(s)—albeit inefficient—may also contribute to its activity. Furthermore, mAb 2C7 that possessed the E430G “complement-enhancing” alteration in Fc was more effective than 2C7 mAb (WT Fc), both in bactericidal assays and in vivo. Strong evidence for the role of complement alone was shown by loss of mAb 2C7 activity in *C9*^*−/−*^ mice. These data also suggest that a “mature,” approximately 10 nm MAC pore that contains 12–18 C9 molecules [32–34] is required for mAb 2C7 efficacy; the approximately 2–3 nm pore formed by C5b-8 [33, 66, 67] is insufficient for killing of gonococci.

Second, our studies also show that Fc-FcγR engagement of 2C7 mAb with phagocytes was not necessary. The K322A variant, whose complement-activating capability was abrogated in the face of preserved Fc-FcγR function, was ineffective in clearing infection in mice, suggesting indirectly that phagocytes were not necessary for this function. Human IgG1 binds to all mouse FcγRs and potently induces Ab-mediated cellular cytotoxicity (ADCC) and Ab-dependent cellular phagocytosis (ADCP) with mouse effector cells in a manner similar to human cells [68]; therefore, we conclude that mouse models are appropriate for evaluating Fc-mediated effects of human IgG1 mAbs. We also blocked complement activation at the level of C5, which abrogated the effect of mAb 2C7 in clearing infection in mice. Anti-C5 mAb BB5.1 blocks generation of C5a and prevents its interactions with C5aR1 that otherwise mediates neutrophil chemotaxis and the oxidative burst [45]; however, C5a–C5aR1 interaction and PMNs are dispensable for mAb 2C7 activity, as discussed below.

Third, depletion of PMNs from mice, using anti-granulocyte receptor-1 (Gr1) mAb RB6-8C5, did not diminish mAb 2C7’s ability to clear infection. This provides additional evidence that PMNs were not necessary to clear infection, thereby supporting mAb 2C7’s role in activating complement as the principal mechanism involved. Furthermore, mAb 2C7’s ability to clear infection in the face of C5a blockade contradicts a role for phagocytes in clearance. Over 80 y ago, Spink and Keefer suggested that phagocytes did not contribute to killing of gonococci in whole blood [69], a finding supported by several more recent studies showing that gonococci have evolved several mechanisms to survive within and among neutrophils (reviewed in [37, 70]).

This study demonstrates the requirement of terminal complement for Ab-mediated clearance of a pathogen at a mucosal surface. It is worth noting that antibodies elicited by meningococcal capsular polysaccharide conjugate vaccines eradicate nasopharyngeal colonization, both in humans and in mouse models [71, 72]. Whether Ab-mediated meningococcal eradication at mucosal surfaces requires complement, phagocytes, or both remains to be determined. Price and Boettcher showed that human cervical mucus collected midcycle possessed 11.5% of complement-mediated hemolytic activity seen in a corresponding volume of undiluted human serum [73]. Complement in genital secretions may originate from plasma exudation during inflammation. Local production of classical and terminal pathway complement components may also contribute significantly to lytic complement activity at mucosal surfaces. Primary human cervical and vaginal epithelial cells can also synthesize all components of the alternative pathway [74]. Several other cell types, including neutrophils and macrophages, produce complement proteins, including terminal pathway components [75, 76]. Taken together, these data support the assumption that complement in the genital tract is present in amounts sufficient to kill gonococci through MAC insertion.

Although a serum bactericidal Ab titer of 1:4 or greater using human complement as the complement source has been well established as the correlate of protection for vaccines against *N*. *meningitidis* [77], lack of knowledge of the correlate(s) of protection against gonococcal infection has posed an obstacle to development of vaccines against gonorrhea. Uncovering the central role for complement and correlating the extent of complement activation with efficacy point to the serum bactericidal assay as a correlate of protection for mAb 2C7. Several potential vaccine candidates that elicit bactericidal antibodies have been identified. The 2C7 LOS epitope that gave rise to 2C7 mAb, reported here, was converted to peptide mimics, identified by screening a random peptide library with mAb 2C7. Screened peptides were down-selected and optimized for antigenicity [15]; one has been formulated into a multiantigenic peptide (MAP). When used as an immunogen in mice, MAP elicited monospecific polyclonal 2C7 antibodies that were bactericidal against gonococci. Mice immunized with the 2C7 peptide mimic cleared vaginal colonization more rapidly and reduce gonococcal burdens in a manner similar to that shown here with mAb 2C7 [16]. A number of other gonococcal surface components that elicit bactericidal antibodies are under examination as vaccine candidates (reviewed in [78]). Prediction of efficacy in vivo by bactericidal Ab activity lends optimism for candidacy of these vaccine antigens.

Several factors contribute to the host restriction of *N*. *gonorrhoeae* infections [79]: these include specificity of gonococcal Opacity proteins (Opa) for human carcinoembryonic antigen cellular adherence molecules (CEACAMs) [80–83], the presence of complement receptor 3 (CR3) on primary human (but not mouse) cervical epithelial cells (CR3 facilitates invasion of gonococci into epithelial cells [84]), the ability to use only human transferrin and lactoferrin as iron sources [85, 86], and the ability to evade human complement [48, 49]. Human-specific complement resistance of gonococci results from selective binding of the human complement inhibitors, FH and C4BP [48, 49]. *N*. *gonorrhoeae*–bound FH and C4BP raise the threshold for bactericidal activity of mAb 2C7 and, as we demonstrated, also raise the threshold for clearance of infection in mice. Sialylation of *N*. *gonorrhoeae*, as occurs in vivo, increases human FH binding [10] and also decreases the bactericidal activity of mAb 2C7 [14], representing another barrier that mAb 2C7 must overcome. In prior studies, we demonstrated that an isogenic gonococcal mutant strain, constructed to bind C4BP, required recruitment of the alternative complement pathway for mAb 2C7 to mediate bactericidal activity against it; however, the parent C4BP nonbinding strain was killed solely by initiating the classical pathway without requiring the alternative pathway [43]. The Ab concentration required for 50% bactericidal activity (BC_50_) of mAb 2C7 is lower for strains that do not bind C4BP compared to the BC_50_ for C4BP-binding strains [43]. Therefore, in studies reported here, it was important to examine mAb 2C7 efficacy in the presence of human C4BP and FH, circumstances that a therapeutic antigonococcal mAb would encounter in vivo. mAb 2C7-E430G Fc was more efficacious than non-Fc variant chimeric mAb 2C7 in clearing infection in FH/C4BP dual Tg mice, although higher doses of mAb 2C7-E430G Fc were required for comparable efficacy compared to doses required in WT mice.

In conclusion, we provide proof of concept that engineering Fc to enhance complement activation—in this case, using HexaBody technology—increases activity of an antibacterial Ab. Fc variants of specific mAbs that enhance effector functions and thereby promote increased bactericidal activity at relatively low concentrations have substantial potential for the treatment of infectious diseases.

## Methods

### Ethics statement

All research involving human participants was approved by the Institutional Review Board (IRB) at the University of Massachusetts. All clinical investigation was conducted according to the principles expressed in the Declaration of Helsinki. Oral informed consent was obtained from the participants in accordance with IRB Protocol #H00013200. Use of animals was performed in strict accordance with recommendations in the *Guide for the Care and Use of Laboratory Animals* of the National Institutes of Health. The protocol (protocol number A-1717) was approved by the IACUC at the University of Massachusetts Medical School.

### Production of chimeric 2C7 mAbs

Sequencing of the V_H_ and V_L_ regions of murine mAb 2C7 specific for the 2C7 LOS epitope [14] was performed on 2C7 hybridoma cells by Genscript (amino acid sequences shown in S1A Fig). Generation of chimeric mAb 2C7 Fc variants (generally referred to as Ab variants below) and their production was performed using the Expi293 expression system (Thermo Fisher Scientific). Briefly, codon-optimized Ab genes encoding heavy and light chains were synthesized (GeneArt, Regensburg, Germany) and cloned separately into pcDNA3.3 (Thermo Fisher Scientific). All Fc domain mutations were introduced in heavy-chain expression vectors either using QuikChange technology (Agilent Technologies, Santa Clara, CA, United States) or via gene synthesis (GeneArt). The mutated positions on Fc are indicated (S1B Fig), and numbering is according to EU nomenclature. Antibodies were expressed in Expi293F cells by transfection of light-chain and heavy-chain expression vector DNA using ExpiFectamine 293 according to manufacturer’s instructions (Thermo Fisher Scientific). Antibodies were purified by Protein A affinity chromatography (rProtein A FF; GE Healthcare, Little Chalfont, Buckinghamshire, United Kingdom), dialyzed overnight against PBS, and filter-sterilized through 0.2 μM dead-end filters (Corning, Amsterdam, the Netherlands). Concentrations of purified IgGs were determined by absorbance at 280 nm (Nanodrop photospectrometer, Thermo Scientific, Breda, the Netherlands) and by using individually predicted extinction coefficients. Quality control (purity, identity, and solubility) of purified antibodies was performed by CE-SDS on the Labchip GXII (Caliper Life Sciences/PerkinElmer Hopkinton, MA, US) under reducing and nonreducing conditions (>90% intact IgG, >95% intact heavy and light chains separately identified under reducing conditions), ESI-TOF MS (Waters, Elstree, Hertfordshire, UK) or Orbitrap (Thermo Scientific), and HP-SEC (aggregate level < 5%) (Waters Alliance 2975 separation unit, Waters) as described previously [19].

### FcγR binding assays

Binding of the purified 2C7-Fc Ab (hu-IgG1) variants to a monomeric extracellular domain (ECD) of FcγRI and to dimeric variants of ECDs of FcγRIIA allotype 131H, FcγRIIA allotype 131R, FcγRIIB, FcγRIIIA allotype 158F, and FcγRIIIA allotype 158V were tested in ELISA assays using the purified Ab variants.

To analyze binding to FcγRI (monomeric ECD), 96-well Microlon ELISA plates (Greiner, Frickenhausen, Germany) were coated with His-tagged FcγRI ECD (1 μg/mL) in PBS, incubated overnight at 4°C, and then washed and blocked with 200 μL/well PBS/0.2% BSA for 1 h at room temperature (RT). After washing, purified mAb 2C7-Fc Ab variants (0.001–10 μg/mL in 10-fold dilutions in PBST/0.2% BSA) were dispensed (100 μL/well of each dilution) to the ECD coated plates for 1 h at RT, the plates were washed, and then detecting Ab (100 μL/well of anti-human-Fcγ-HRP [Jackson, 109-035-098, 1:5,000] in PBST/0.2% BSA) was added and incubated at RT for 30 min. As indicated, washing was performed after each incubation step. Bound conjugate was disclosed with 1 mg/mL 2,2′-azino-bis (3-ethylbenzothiazoline-6-sulfonic acid) (ABTS; Roche, Mannheim, Germany) for approximately 20 min and reactions stopped by adding 100 μL 2% oxalic acid.

To analyze binding to dimeric FcγR variants [87], 96-well Microlon ELISA plates (Greiner, Germany) were coated with goat F(ab’)_2_-anti-human-IgG-F(ab’)_2_ (Jackson Laboratory, 109-006-097, 1 μg/mL) in PBS, washed and blocked with 200 μL/well PBS/0.2% BSA for 1 h at RT, and incubated overnight at 4°C. After washing, purified mAb 2C7-Fc Ab variants (0.001–10 μg/mL in 10-fold dilutions in PBST/0.2% BSA) were dispensed (100 μL/well of each dilution) to the goat F(ab’)_2_-anti-human-IgG-F(ab’)_2_ coated plates and incubated for 1 h at RT. After washing, 100 μL/well of dimeric, His-tagged, C-terminally biotinylated FcγR ECD variants (1 μg/mL) in PBST/0.2% BSA was added for 1 h at RT. Again, after washing, 100 μL/well Streptavidin-polyHRP (CLB, M2032, 1:10,000) in PBST/0.2% BSA was added as detecting Ab for 30 min at RT. As indicated, plates were washed between each incubation step. Bound Streptavidin-polyHRP was revealed with 1 mg/mL ABTS (Roche, Mannheim, Germany); reactions were stopped by adding 100 μL 2% oxalic acid after about 15 min (for IIA-131H, IIA-131R, IIIA-158F, IIIA-158V) or 30 min (for IIB).

To establish uniform capture of all Fc variant antibodies, 96-well Microlon ELISA plates were directly coated and incubated with dilutions of 2C7-Fc Ab variants. For detection, plates coated with dilutions of the Ab variants were incubated with 100 μL/well of anti-human-Fcγ-HRP (Jackson, 109-035-098, 1:5,000) in PBST/0.2% BSA for 30 min at RT. Bound HRP-conjugate was disclosed with ABTS as described above; color reactions were stopped at about 10 min by adding 100 μL 2% oxalic acid.

In the ELISA binding assays above, absorbance was measured at 405 nm in a microplate reader (BioTek, Winooski, VT, US). Log-transformed data were analyzed by fitting sigmoidal dose-response curves with variable slope using GraphPad Prism 7.02 software. The area under the dose-response curve was calculated using a log-transformed concentration axis.

### Bacterial strains

*N*. *gonorrhoeae* strains FA1090 [88], MS11 [89], and 15253 [24] and *C*. *muridarum* [90] have been described previously. All three gonococcal strains used in this study are piliated (Pil^+^) and express Opa (Opa^+^). Strains FA1090 and MS11 are naturally resistant to streptomycin (Sm; used in experimental animals and culture media to reduce competitive microflora [see below]). Strain 15253 was transformed with the rpsL gene from Sm-resistant strain FA1090 [56].

### Flow cytometry

Binding of mAb 2C7 to *N*. *gonorrhoeae* by flow cytometry was performed as described previously [23]. Briefly, increasing concentrations of each of the mAb 2C7 preparations were incubated with bacteria for 15 min at 37°C and washed twice, and bound mAb 2C7 was detected with FITC-conjugated anti-human IgG specific for the Fc region (Sigma).

### Human serum

Serum was obtained from normal healthy adult volunteers (NHS) who had no prior history of gonococcal or meningococcal infection and who provided informed consent. Serum was obtained from whole blood that was clotted for 25°C for 30 min, followed by centrifugation at 1,500*g* for 20 min at 4°C. Sera from 10 individuals were pooled and stored at −70°C. Serum bactericidal assays with strain MS11 were performed with human complement (NHS depleted of IgG and IgM [Pel-Freez]) to eliminate background killing by NHS alone.

### Serum bactericidal assay

Serum bactericidal assays were performed as described previously [16]. Bacteria harvested from overnight cultures were repassaged on chocolate agar, grown for 6 h, and suspended in HBSS containing 0.15 mM CaCl_2_ and 1 mM MgCl_2_ (HBSS^++^). About 2,000 CFU of suspended bacteria were incubated with NHS and mAb 2C7 (concentration specified for each experiment). The final reaction volumes were maintained at 150 μl. Aliquots of 25 μl of reaction mixtures were plated onto chocolate agar in duplicate at the beginning of the assay (t_0_) and again after incubation at 37°C for 30 min (t_30_). Survival was calculated as the number of viable colonies at t_30_ relative to t_0_.

### Whole-cell ELISA for C1q binding and C4 and C3 deposition

Whole-cell ELISA for complement component deposition was performed using previously described methods [91]. Briefly, 10^5^ CFU of strain FA1090 in HBSS^++^ were incubated with 16.7% human complement (Pel-Freez) in a final volume of 600 μl for 30 min at 37°C. Bacteria were washed in HBSS at 4°C and allowed to coat microtiter wells for 3 h at 37°C. Bacteria-bound C1q, C3, and C4 were detected using goat polyclonal antisera against each of these complement proteins (Complement Technologies), followed by anti-goat IgG conjugated with alkaline phosphatase (Sigma).

### Mouse strains

WT BALB/cJ mice were purchased from the Jackson Laboratory. C1q^*−/−*^ mice in a C57BL/6 background have been described previously [92]. Human FH and C4BP dual Tg mice (FH/C4BP Tg) in a BALB/c background were generated at the Transgenic Core Facility at the University of Massachusetts and have been characterized and described previously [47]. *C9*^*−/−*^ mice in a C57BL/6 background were generated as described previously; these mice lack C9 protein expression and lack serum hemolytic activity [93].

### Mouse infection studies

A consolidated summary of the different procedures used is shown in S8 Fig, indicating the different schedules used when different routes of administration of mAb 2C7/Ab variants or different interventions (coinfection with *C*. *muridarum*, PMN depletion, or C5 blockade [see below]) were employed. Female BALB/c mice 6–8 wk of age (The Jackson Laboratory) in the diestrus phase of the estrous cycle were started on treatment (that day) with 0.5 mg Premarin (Pfizer) in 200 μl water given subcutaneously on each of 3 d; −2, 0, and +2 d (before, the day of, and after inoculation) to prolong the estrus phase of the cycle and promote susceptibility to *N*. *gonorrhoeae* infection [94]. Premarin is a mixture of sodium estrone sulfate and sodium equilin sulfate and as concomitant components, sodium sulfate conjugates of 17α-dihydroequilin, 17α-estradiol, and 17β-dihydroequilin. Mice were administered vancomycin (0.6 mg) and Sm sulfate (0.3 mg) intraperitoneally on each of 3 d; −2, −1, and 0 d (before and the day of inoculation) to reduce competitive microflora [94]. The inoculum size was specified for each experiment. Daily bacterial burdens were measured by enumerating CFU obtained by first ringing vaginal swabs in 100 μl of normal saline and then plating serial 10-fold dilutions onto chocolate agar plates containing Isolivitalex equivalent and VCNTS (vancomycin, colistin, nystatin, and trimethoprim sulfate) supplement (Becton Dickinson, Cockeysville, MD, US) plus 100 mg of Sm sulfate (Sigma, St. Louis, MO, US) per ml of media [94].

The efficacy of intravaginally administered mAb 2C7 or Ab variants was examined by administering intravaginally (daily, for the duration of the experiment) saline (vehicle control) or varying doses (0.1, 0.5, 1, or 10 μg) of 2C7 mAbs or Ab variants; daily bacterial burdens were determined. The limit of detection was 4 CFU per swab eluted in 100 μl saline. Analyses of the data are described below. Clearance of infection was defined by 3 or more consecutive days of negative cultures.

The efficacy of systemically administered mAb 2C7-E430G was studied by injecting the mAb IV through the tail vein at a single dose of 10 μg or 100 μg on day 1. Gonococcal CFUs were enumerated daily by vaginal swabbing as described above.

To compare the efficacy of mAb 2C7 and mAb 2C7-E430G in the context of the human complement inhibitors, we used Tg mice that expressed human FH and C4BP. These mice express complement inhibitor levels that are similar to that seen in NHS and have normal inflammatory responses to a variety of inflammatory stimuli [47]. Mice were infected and treated daily intravaginally as described above for WT BALB/c mice.

The *N*. *gonorrhoeae–C*. *muridarum* coinfection model was performed as described previously [55]. Human FH/C4BP Tg mice were infected intravaginally with 2 × 10^6^ IFU *C*. *muridarum* on days −4, −3, and −2. Mice in the diestrus phase of the estrous cycle on day −2 were treated with Premarin and infected with *N*. *gonorrhoeae*, as described above.

### PMN depletion and C5 and C5aR1 blockade

Depletion of mouse PMNs was carried out by intraperitoneal treatment with anti-Gr1 mAb RB6-8C5 (rat IgG2b; BioXCell; [95, 96]) at a dosage of 200 μg on day 0 and 100 μg on day +3. Anti-KLH mAb LTF-2 (rat IgG2b) was used as the isotype control. Depletion of PMNs in mice treated with RB6-8C5 was confirmed by complete peripheral blood counts, which showed <100 neutrophils/mm^3^ up to day 6 after administration of anti-Gr1 mAb RB6-8C5. Giemsa-stained smears obtained by vaginal swabbing also confirmed the absence of PMNs in vaginal secretions up to day 7 in mAb RB6–treated animals (S5 Fig). Mouse C5 function in vivo was blocked using mAb BB5.1 [97] (kindly provided by Dr. B. Stockinger) that was purified by affinity chromatography over protein A agarose from tissue culture supernatants at the University of Virginia’s Antibody Engineering and Technology core facility. Mice were administered 1 mg mAb BB5.1 intraperitoneally on days −1, 2, and 5; in addition, mice were given 5 μg of mAb BB5.1 daily intravaginally to block locally produced C5. The selective C5aR1 antagonist PMX205 was synthesized as described previously [98, 99]. PMX205 is a small cyclic peptide (hydrocinnamate-[Orn-Pro-dCha-Trp-Arg]) that has nanomolar potency as a noncompetitive selective inhibitor of C5aR1 [99, 100]. PMX205 lacks activity in *C5aR1*^*−/−*^ mice, demonstrating specificity [101]. Mice were given 3 μg/kg (75 μg/dose) of PMX205 in PBS subcutaneously every other day from day −2 to day +6 and were also administered 5 μg of the drug daily intravaginally from day 0 to day 8 to block C5aR1.

### Statistical analyses

Differences between the bactericidal efficacy and the complement binding abilities of mAb 2C7 E430G Fc and WT Fc at various concentrations were assessed by two-way ANOVA. Experiments that compared clearance of *N*. *gonorrhoeae* in independent groups of mice estimated and tested three characteristics of the data [16, 91]: time to clearance, longitudinal trends in mean log_10_ CFU, and the cumulative CFU as AUC. Median time to clearance was estimated using Kaplan Meier survival curves; times to clearance were compared between groups using the Mantel-Cox log-rank test. Significance was set using Bonferroni correction when more than two groups were compared and is indicated for each experiment. The mean AUC (log_10_ CFU versus time) was computed for each mouse to estimate the bacterial burden over time (cumulative infection); the means under the curves were compared between groups using the Kruskal-Wallis nonparametric rank-sum test because distributions were skewed or kurtotic. Pairwise comparisons between groups were performed using Dunn’s post hoc test.

## Supporting information

S1 Fig (A) Schematic of *N*. *gonorrhoeae* LOS. The phase-variable *lgt* genes are indicated in black boxes. The LOS structure required for mAb 2C7 binding is indicated in the blue shaded box. V_H_ and V_L_ (lambda) sequences of mAb 2C7 are shown. (B) Schematic of chimeric mAb 2C7 molecules showing location of the Fc variations. (C) Binding to *N*. *gonorrhoeae* strain FA1090 of mAb 2C7 (WT Fc) and 2 Fc derivatives (mAbs 2C7- E430G Fc and 2C7-D270A/K322A Fc). Data associated with this figure can be found in the supplemental data file (S1 Data). *lgt*, LOS glycosyltransferase; LOS, lipooligosaccharide; mAb, monoclonal antibody; V_H_, variable domain, heavy chain; V_L_, variable domain, light chain; WT, wild-type.
(TIF)

S2 Fig Increased efficacy of mAb 2C7 E430G Fc (abbreviated E430G Fc) compared to mAb 2C7 (WT Fc) against *N. gonorrhoeae* FA1090 in the mouse vaginal colonization model. WT BALB/c mice were infected with 8.75 × 10^5^ CFU *N*. *gonorrhoeae* FA1090 and treated intravaginally (daily, for 10 d) with mAbs 2C7-E430G Fc and 2C7-WT Fc (each mAb at doses of 1, 0.5 or 0.1 μg/d), the “complement inactive” mAb 2C7-D270A/K322A Fc variant (abbreviated D270A/K322A Fc) at 10 μg/d, or saline (vehicle control). Vaginal *N*. *gonorrhoeae* CFU were enumerated daily. (A) Kaplan Meier graph shows time to clearance of infection. Significance was set at 0.0018 (Bonferroni correction for eight groups). The table on the right shows a pairwise comparison between the curves. (B) Bacterial burdens (expressed as log_10_ CFU) over time (mean [SEM]). (C) AUC analysis. The median and 95% confidence interval are shown for each group. Comparison across the groups by one-way ANOVA were significant (*P* < 0.0001; Kruskal-Wallis test). The table on the right shows the relevant pairwise comparisons between groups using Dunn’s post hoc test. Data associated with this figure can be found in the supplemental data file (S1 Data). AUC, area under curve; CFU, colony-forming units; mAb, monoclonal antibody; WT, wild-type.
(TIF)

S3 Fig Binding of mAb 2C7 (WT Fc) and mAb 2C7 Fc variants to six FcγRs displayed as AUC. Area under the dose-response curve taken from data shown in Fig 3 was calculated using a log_10_ transformed concentration axis. The mean (SEM) of three separate experiments is shown. Data associated with this figure can be found in the supplemental data file (S1 Data; AUC were calculated from the raw data for Fig 3 using GraphPad Prism). AUC, area under curve; CFU, colony-forming units; FcγR, Fc gamma receptor; mAb, monoclonal antibody; WT, wild-type.
(TIF)

S4 Fig C5 blockade abrogates efficacy of systemically administered mAb 2C7-E430G Fc. C5 function in wild-type BALB/c mice was blocked with mAb BB5.1 (1 mg intraperitoneally on days −1, 2, and 5). In addition, 10 μg of mAb BB5.1 was also administered intravaginally (daily for 8 d, beginning on day 1). Mouse IgG1 was used as a control in mice not given mAb BB5.1. Four groups of mice (*n* = 5/group) treated as follows were infected with *N*. *gonorrhoeae* FA1090 (3.6 × 10^7^ CFU): (1) wild-type mice given control mouse IgG1; (2) C5 blockade with mAb BB5.1; (3) wild-type mice given control mouse IgG1, treated with mAb 2C7-E430G Fc (0.5 μg intravaginally from days 1 through 8); and (4) C5 blockade with mAb BB5.1, treated with mAb 2C7-E430G Fc 0.5 μg intravaginally from days 1 through 8. Left graph: Kaplan Meier graph showing time to clearance of infection. Significance was set at 0.008 (Bonferroni correction for 4 groups). *P* = 0.0016 for mice given control mouse IgG1 and treated with E430G versus all other groups. Middle graph: Log_10_ CFU versus time (mean [SEM]). Right graph: AUC analysis. The median and 95% confidence intervals are shown for each group. Comparison across groups by one-way ANOVA by Kruskal-Wallis test showed significance (*P* = 0.0103). Pairwise comparisons across groups were made with Dunn’s post hoc test. Data associated with this figure can be found in the supplemental data file (S1 Data). AUC, area under curve; CFU, colony-forming units; IgG1, immunoglobulin G1; mAb, monoclonal antibody.
(TIF)

S5 Fig Enumeration of PMNs in mouse vaginal secretions to ensure PMN depletion. The vaginas of mice infected with *N*. *gonorrhoeae* and treated with rat IgG2b (isotype control for mAb RB6) and no 2C7 (saline as a vehicle control; blue triangles), rat IgG2b followed by mAb 2C7 (green inverted triangles), RB6 and saline (open gray circles), or mAb RB6 followed by mAb 2C7 (red squares) were swabbed, cells were fixed on a slide and stained with Giemsa stain, and the percentage of PMNs among 100 counted cells was enumerated. The graph to the left shows PMNs as percentage of all counted cells (mean, data for each individual mouse indicated), and the graph to the right shows the percentage of mice in each group with PMNs detected in the vagina. Data associated with this figure can be found in the supplemental data file (S1 Data). IgG2b, immunoglobulin G2b; mAb, monoclonal antibody; PMN, polymorphonuclear neutrophil.
(TIF)

S6 Fig FA1090 infection in human FH/C4BP dual Tg mice (shown in S2 Fig as saline [control]) compared to infection in WT BALB/c mice (shown in Fig 7 as saline [control]) show increased duration and burden of gonococcal infection. The bacterial burdens in the saline control administered WT mice (S2 Fig) and human FH/C4BP dual Tg mice (Fig 7) are compared. Experiments in which results are shown in S2 and S7 Figs were performed at the same time; mice were challenged simultaneously with 8.75 × 10^5^ CFU *N*. *gonorrhoeae* FA1090. Vaginal CFUs were enumerated daily for 10 d. (A) Kaplan Meier graph shows time to clearance of infection (comparison by Mantel-Cox log-rank test). (B) Bacterial burdens (expressed as log_10_ CFU) over time (mean [SEM]). (C). AUC analysis (median with 95% confidence interval) is shown. The two groups were compared using Mann-Whitney’s nonparametric test. Data associated with this figure can be found in the supplemental data file (S1 Data). AUC, area under curve; C4BP, C4b-binding protein; CFU, colony-forming units; FH, factor H; Tg, transgenic; WT, wild-type.
(TIF)

S7 Fig mAb 2C7-E430G Fc (abbreviated as E430G) is efficacious against additional strains of *N. gonorrhoeae* (strains MS11 and 15253) in human FH/C4BP dual Tg mice. FH/C4BP Tg mice were infected with 3.4 × 10^5^ CFU strain MS11 (A-C) or 7.3 × 10^5^ CFU strain 15253 (D-F). (A and D) Kaplan Meier graphs showing time to clearance of infection. Tables beneath each graph show groups that differed significantly by Mantel-Cox log-rank test. Significance set at 0.005 (Bonferroni correction for 5 groups). (B and E) Bacterial burdens (expressed as log_10_ CFU) over time (mean [SEM]). (C and F) AUC analysis. The median and 95% confidence interval are indicated for each group. Comparison across groups made by one-way ANOVA were significant (*P* < 0.0001; Kruskal-Wallis test). Pairwise comparisons between groups were performed using Dunn’s post hoc test. *P < 0.05; ****P < 0.0001. Data associated with this figure can be found in the supplemental data file (S1 Data). AUC, area under curve; C4BP, C4b-binding protein; CFU, colony-forming units; FH, factor H; mAb, monoclonal antibody; Tg, transgenic.
(TIF)

S8 Fig Consolidated summary of the experimental procedures used in mouse experiments, also indicating the different schedules used when different routes of administration of mAb 2C7 antibody/antibody variants or different interventions (coinfection with *C. muridarum*, PMN depletion, C5aR1 or C5 blockade) were employed. CBC, complete peripheral blood count; mAb, monoclonal antibody; *Ng*, *N*. *gonorrhoeae*; PMN, polymorphonuclear neutrophil.
(TIF)

S1 Data Excel spreadsheet containing, in separate sheets, the underlying numerical data and statistical analysis for Figs 1A, 1B, 2A-2C, 2D-2F, 3, 4, 5A, 5B, 5C, 6A, 6B, 6C, 7A-7C, 8A-8C, 8D-8F, 9, S1C, S2A–S2C, S4, S5, S6, S7A–S7C and S7D–S7F. (XLSX)

## Abbreviations

Ab: antibody
ADCC: Ab-mediated cellular cytotoxicity
ADCP: Ab-dependent cellular phagocytosis
AUC: area under curve
C4BP: C4b-binding protein
C5aR1: C5a receptor 1
CEACAM: carcinoembryonic antigen cellular adherence molecule
CFU: colony-forming units
CR3: complement receptor 3
DGI: disseminated gonococcal infection
FcγR: Fc gamma receptor
FH: factor H
Gr1: granulocyte receptor-1
HepI: heptose I
IgG: immunoglobulin G
IV: intravenously
lgtG: LOS glycosyltransferase G
LOS: lipooligosaccharide
mAb: monoclonal Ab
MAC: membrane attack complex
MAP: multiantigenic peptide
NHS: normal human serum
NS: not significant
OD: optical density
Opa: opacity protein
PMN: polymorphonuclear neutrophil
PorB: Porin B
Sm: streptomycin
STI: sexually transmitted infection
Tg: transgenic
V_H_: variable domain, heavy chain
V_L_: variable domain, light chain
WT: wild-type
