# Supplementary figures and images for: Complement alone drives efficacy of a chimeric antigonococcal monoclonal antibody
Source: PLoS Biol. 2019 Jun 19;17(6):e3000323. doi: 10.1371/journal.pbio.3000323 (PMC6602280; doi:10.1371/journal.pbio.3000323)

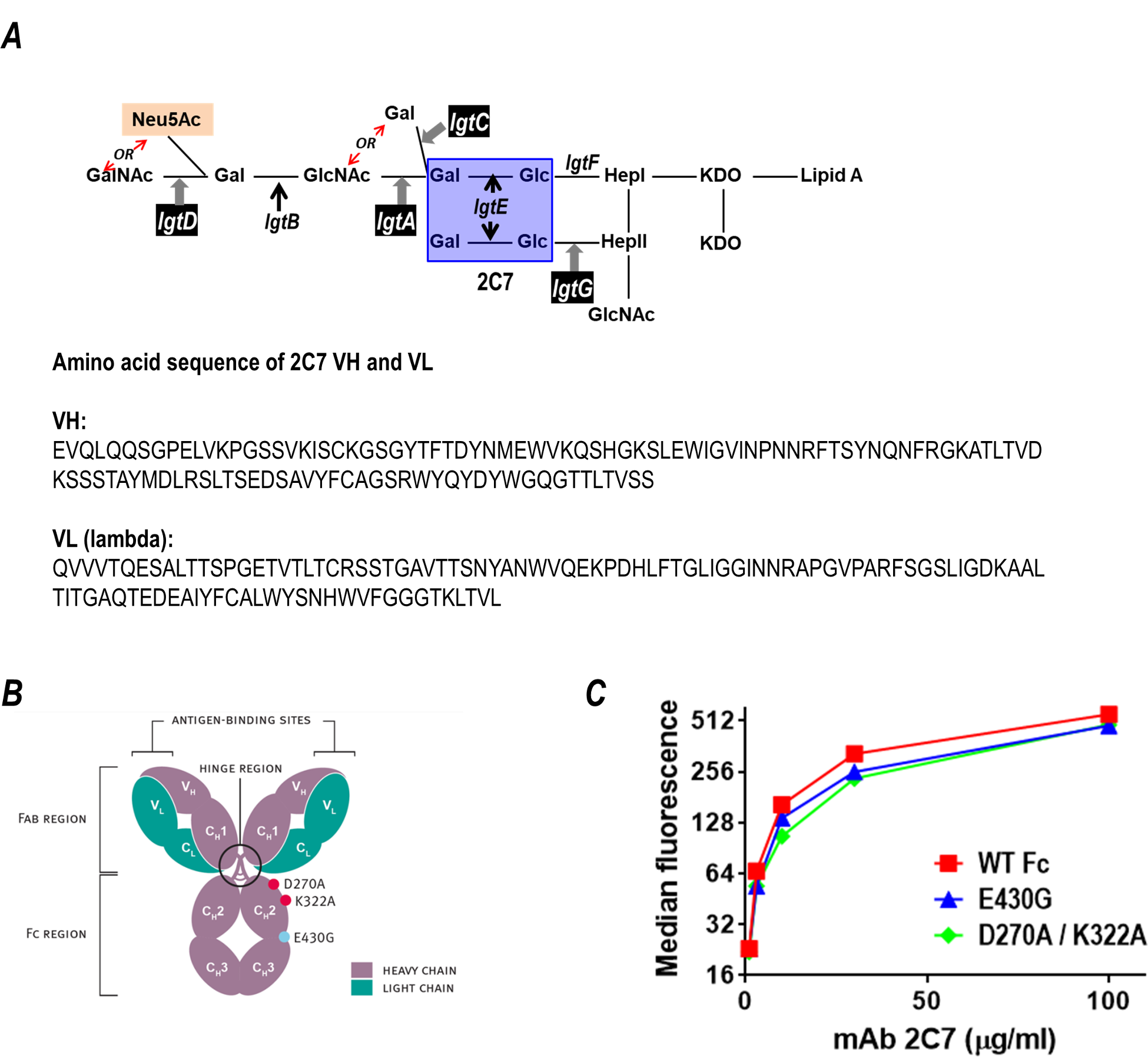

Supplement: S1 Fig — (A) Schematic of N. gonorrhoeae LOS. The phase-variable lgt genes are indicated in black boxes. The LOS structure required for mAb 2C7 binding is indicated in the blue shaded box. VH and VL (lambda) sequences of mAb 2C7 are shown. (B) Schematic of chimeric mAb 2C7 molecules showing location of the Fc variations. (C) Binding to N. gonorrhoeae strain FA1090 of mAb 2C7 (WT Fc) and 2 Fc derivatives (mAbs 2C7- E430G Fc and 2C7-D270A/K322A Fc). Data associated with this figure can be found in the supplemental data file (S1 Data). lgt, LOS glycosyltransferase; LOS, lipooligosaccharide; mAb, monoclonal antibody; VH, variable domain, heavy chain; VL, variable domain, light chain; WT, wild-type. (TIF) [file pbio.3000323.s001.TIF]

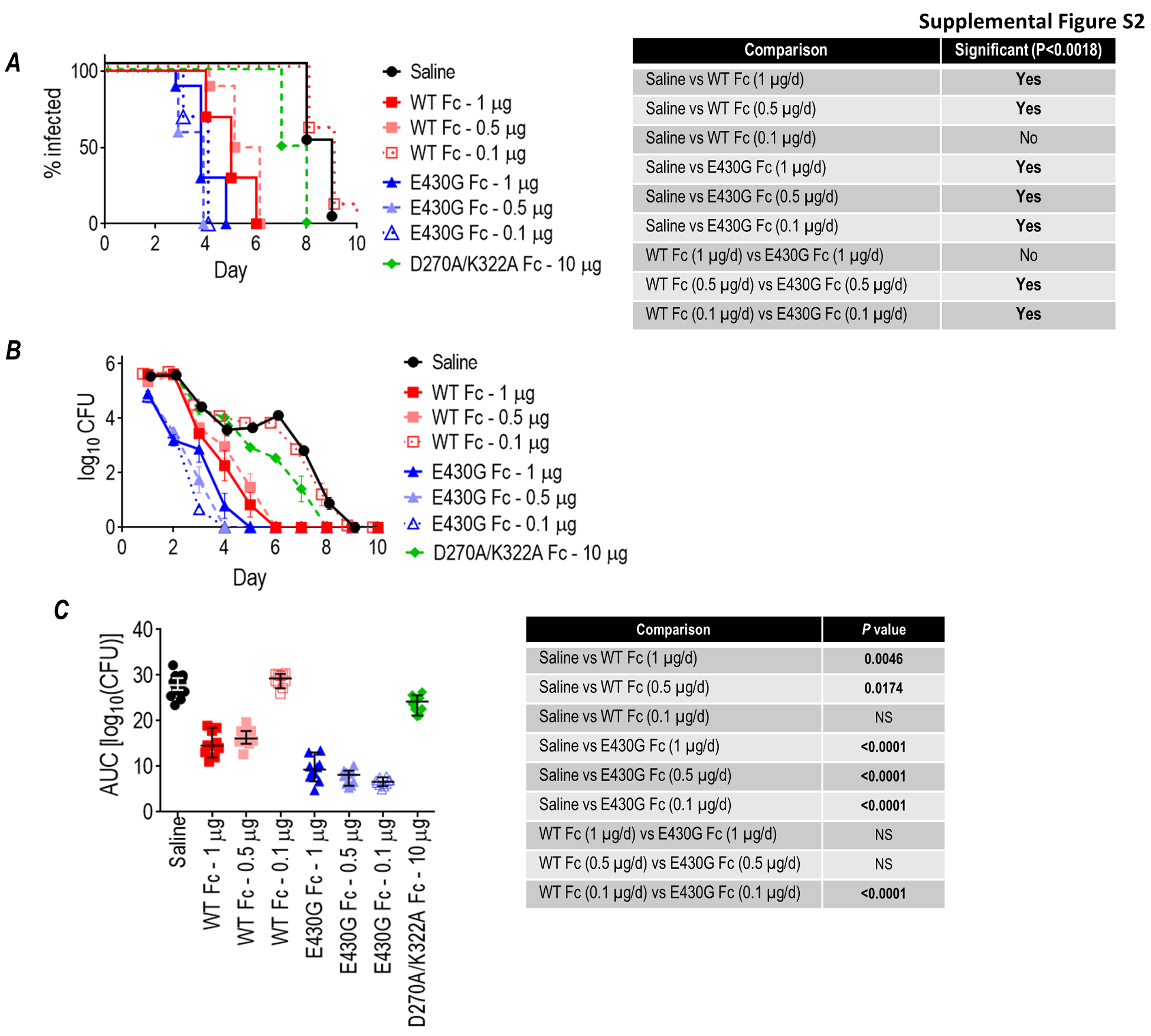

Supplement: S2 Fig — WT BALB/c mice were infected with 8.75 × 10^5^ CFU N. gonorrhoeae FA1090 and treated intravaginally (daily, for 10 d) with mAbs 2C7-E430G Fc and 2C7-WT Fc (each mAb at doses of 1, 0.5 or 0.1 μg/d), the “complement inactive” mAb 2C7-D270A/K322A Fc variant (abbreviated D270A/K322A Fc) at 10 μg/d, or saline (vehicle control). Vaginal N. gonorrhoeae CFU were enumerated daily. (A) Kaplan Meier graph shows time to clearance of infection. Significance was set at 0.0018 (Bonferroni correction for eight groups). The table on the right shows a pairwise comparison between the curves. (B) Bacterial burdens (expressed as log10 CFU) over time (mean [SEM]). (C) AUC analysis. The median and 95% confidence interval are shown for each group. Comparison across the groups by one-way ANOVA were significant (P < 0.0001; Kruskal-Wallis test). The table on the right shows the relevant pairwise comparisons between groups using Dunn’s post hoc test. Data associated with this figure can be found in the supplemental data file (S1 Data). AUC, area under curve; CFU, colony-forming units; mAb, monoclonal antibody; WT, wild-type. (TIF) [file pbio.3000323.s002.TIF]

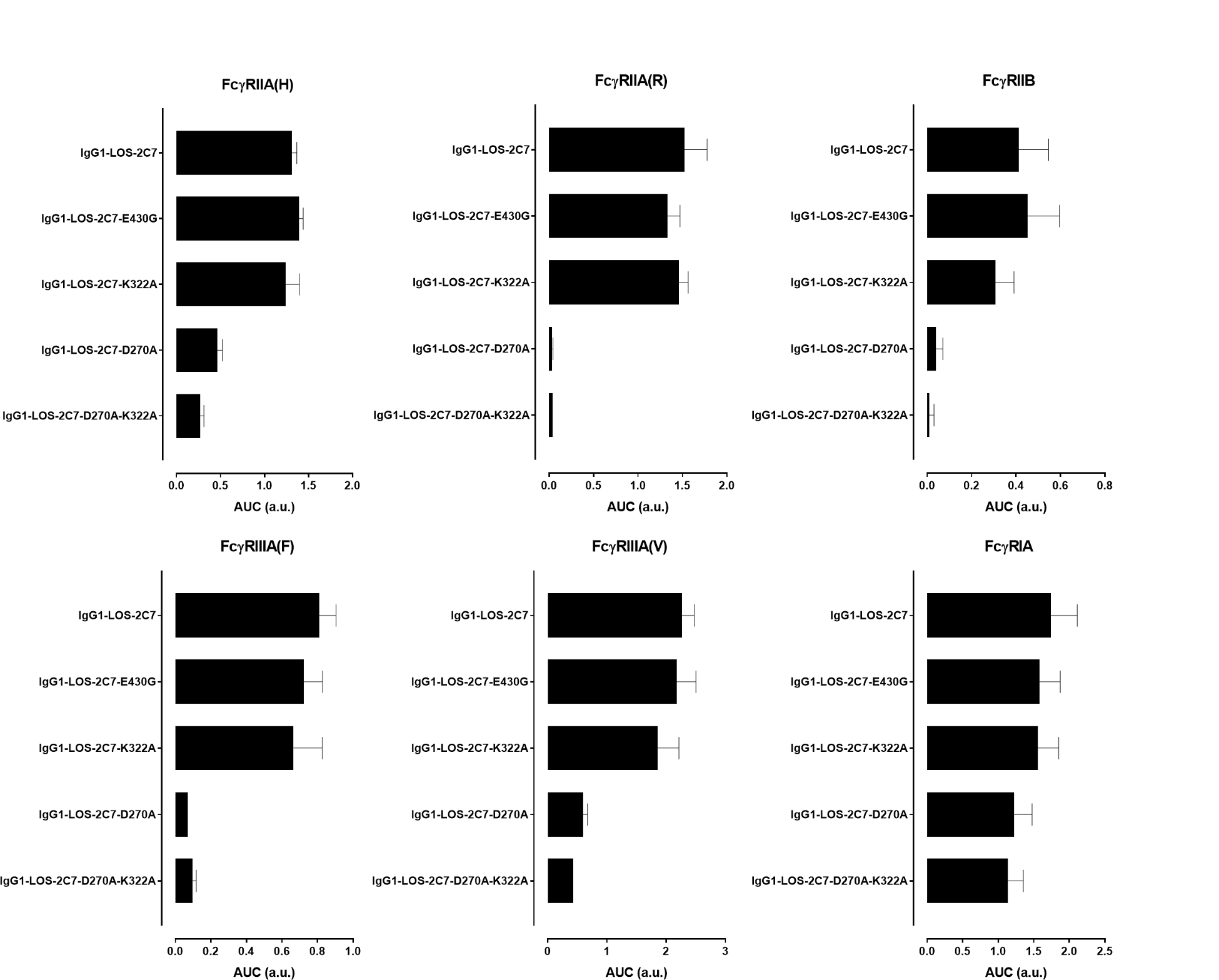

Supplement: S3 Fig — Area under the dose-response curve taken from data shown in Fig 3 was calculated using a log10 transformed concentration axis. The mean (SEM) of three separate experiments is shown. Data associated with this figure can be found in the supplemental data file (S1 Data; AUC were calculated from the raw data for Fig 3 using GraphPad Prism). AUC, area under curve; CFU, colony-forming units; FcγR, Fc gamma receptor; mAb, monoclonal antibody; WT, wild-type. (TIF) [file pbio.3000323.s003.TIF]

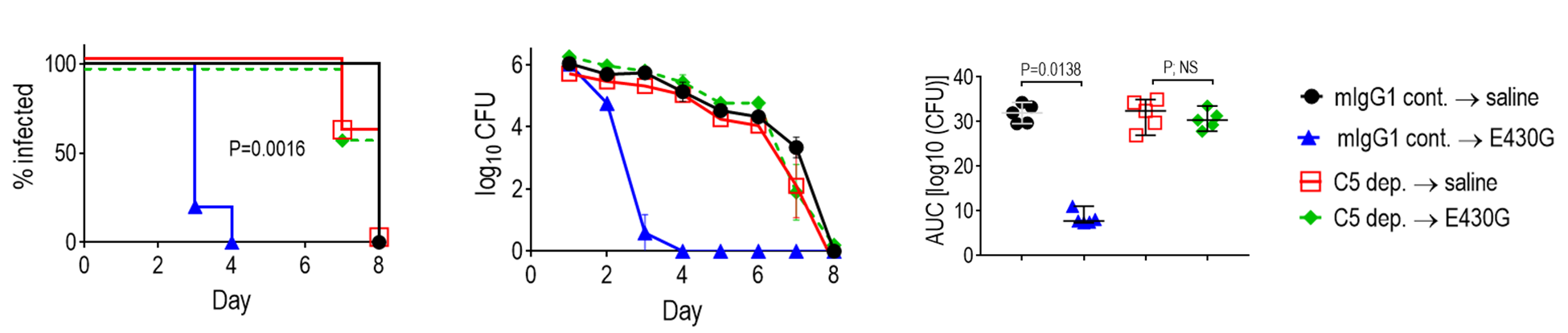

Supplement: S4 Fig — C5 function in wild-type BALB/c mice was blocked with mAb BB5.1 (1 mg intraperitoneally on days −1, 2, and 5). In addition, 10 μg of mAb BB5.1 was also administered intravaginally (daily for 8 d, beginning on day 1). Mouse IgG1 was used as a control in mice not given mAb BB5.1. Four groups of mice (n = 5/group) treated as follows were infected with N. gonorrhoeae FA1090 (3.6 × 10^7^ CFU): (1) wild-type mice given control mouse IgG1; (2) C5 blockade with mAb BB5.1; (3) wild-type mice given control mouse IgG1, treated with mAb 2C7-E430G Fc (0.5 μg intravaginally from days 1 through 8); and (4) C5 blockade with mAb BB5.1, treated with mAb 2C7-E430G Fc 0.5 μg intravaginally from days 1 through 8. Left graph: Kaplan Meier graph showing time to clearance of infection. Significance was set at 0.008 (Bonferroni correction for 4 groups). P = 0.0016 for mice given control mouse IgG1 and treated with E430G versus all other groups. Middle graph: Log10 CFU versus time (mean [SEM]). Right graph: AUC analysis. The median and 95% confidence intervals are shown for each group. Comparison across groups by one-way ANOVA by Kruskal-Wallis test showed significance (P = 0.0103). Pairwise comparisons across groups were made with Dunn’s post hoc test. Data associated with this figure can be found in the supplemental data file (S1 Data). AUC, area under curve; CFU, colony-forming units; IgG1, immunoglobulin G1; mAb, monoclonal antibody. (TIF) [file pbio.3000323.s004.tif]

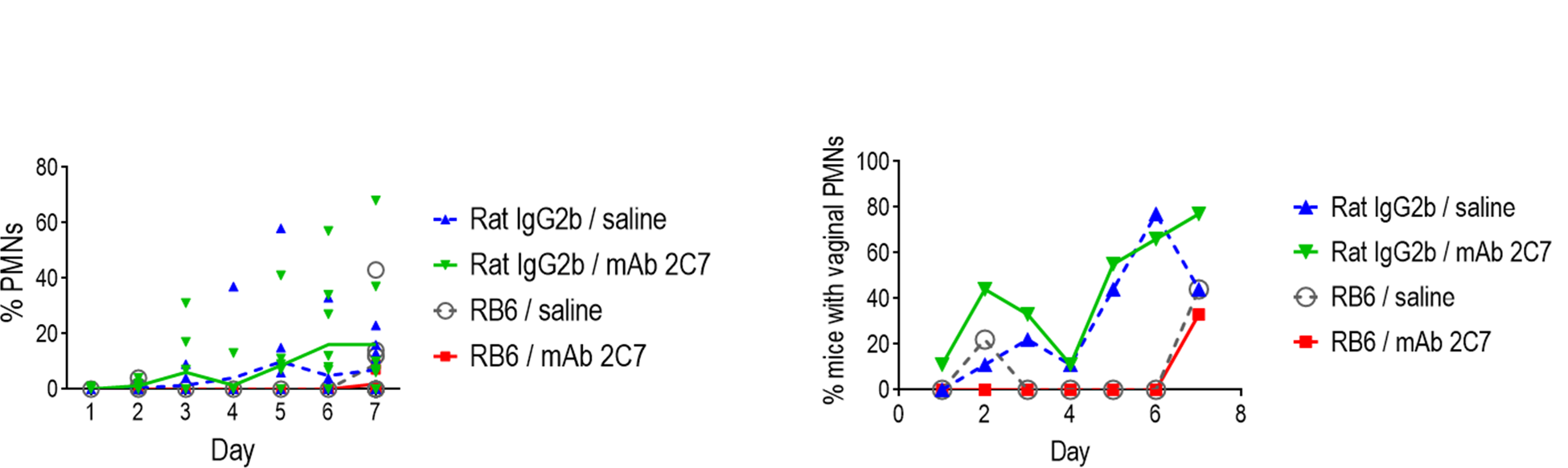

Supplement: S5 Fig — The vaginas of mice infected with N. gonorrhoeae and treated with rat IgG2b (isotype control for mAb RB6) and no 2C7 (saline as a vehicle control; blue triangles), rat IgG2b followed by mAb 2C7 (green inverted triangles), RB6 and saline (open gray circles), or mAb RB6 followed by mAb 2C7 (red squares) were swabbed, cells were fixed on a slide and stained with Giemsa stain, and the percentage of PMNs among 100 counted cells was enumerated. The graph to the left shows PMNs as percentage of all counted cells (mean, data for each individual mouse indicated), and the graph to the right shows the percentage of mice in each group with PMNs detected in the vagina. Data associated with this figure can be found in the supplemental data file (S1 Data). IgG2b, immunoglobulin G2b; mAb, monoclonal antibody; PMN, polymorphonuclear neutrophil. (TIF) [file pbio.3000323.s005.TIF]

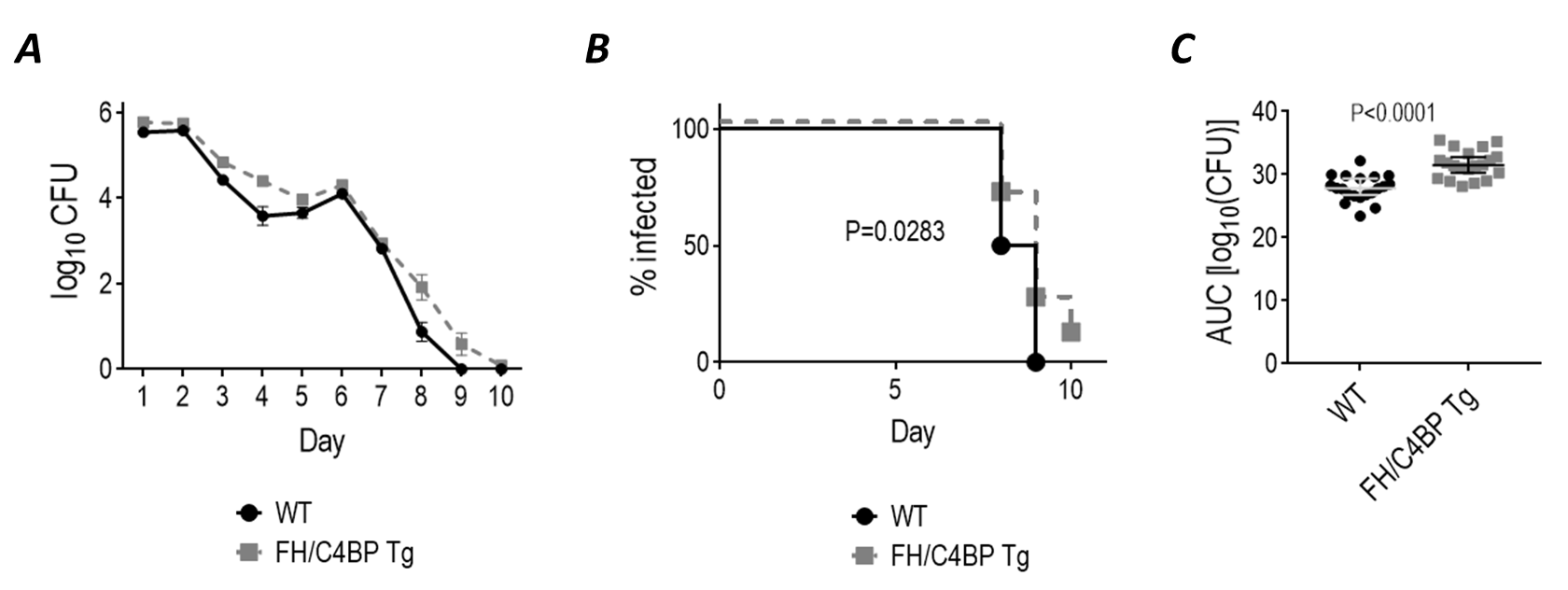

Supplement: S6 Fig — The bacterial burdens in the saline control administered WT mice (S2 Fig) and human FH/C4BP dual Tg mice (Fig 7) are compared. Experiments in which results are shown in S2 and S7 Figs were performed at the same time; mice were challenged simultaneously with 8.75 × 10^5^ CFU N. gonorrhoeae FA1090. Vaginal CFUs were enumerated daily for 10 d. (A) Kaplan Meier graph shows time to clearance of infection (comparison by Mantel-Cox log-rank test). (B) Bacterial burdens (expressed as log10 CFU) over time (mean [SEM]). (C). AUC analysis (median with 95% confidence interval) is shown. The two groups were compared using Mann-Whitney’s nonparametric test. Data associated with this figure can be found in the supplemental data file (S1 Data). AUC, area under curve; C4BP, C4b-binding protein; CFU, colony-forming units; FH, factor H; Tg, transgenic; WT, wild-type. (TIF) [file pbio.3000323.s006.tif]

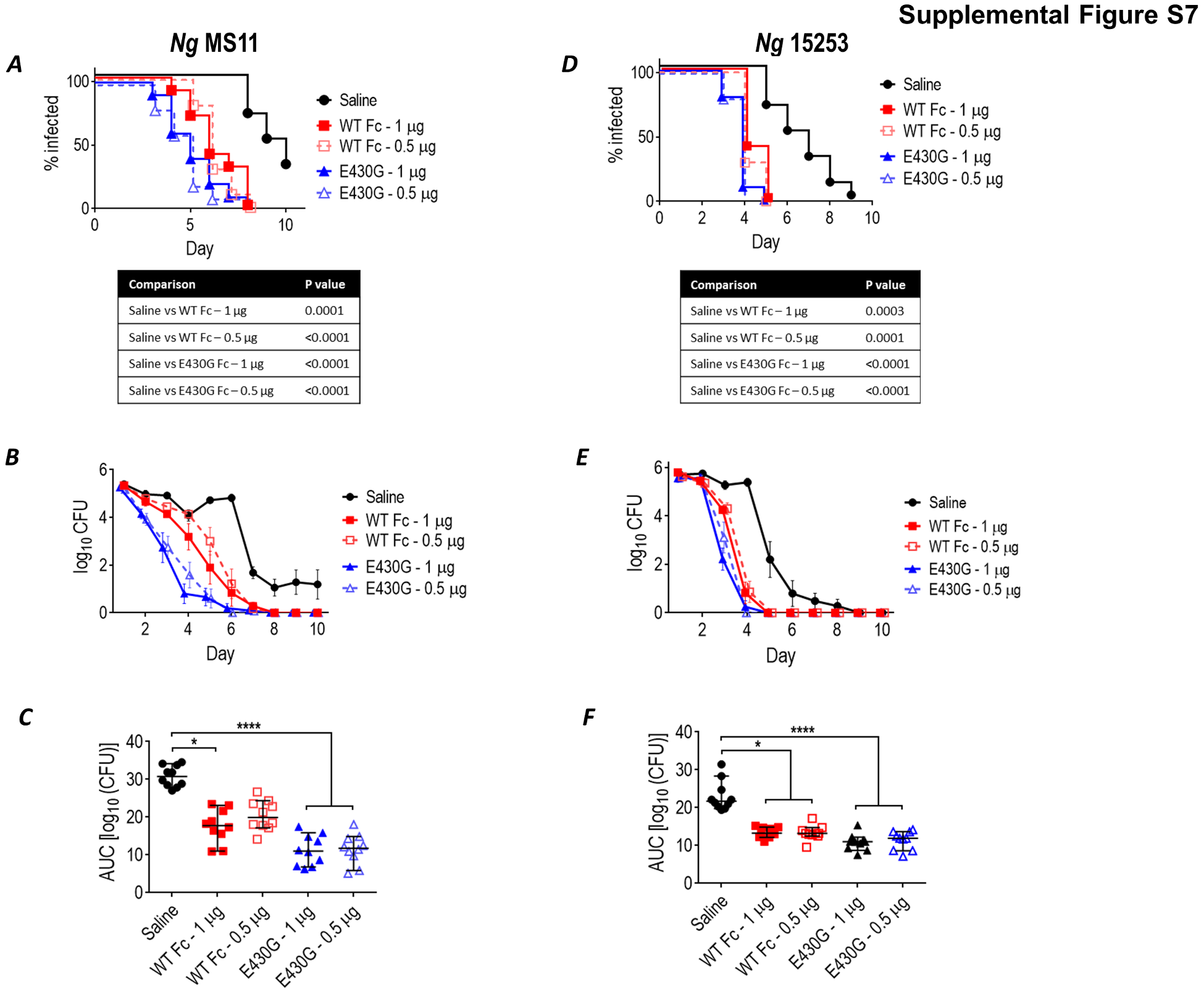

Supplement: S7 Fig — FH/C4BP Tg mice were infected with 3.4 × 10^5^ CFU strain MS11 (A-C) or 7.3 × 10^5^ CFU strain 15253 (D-F). (A and D) Kaplan Meier graphs showing time to clearance of infection. Tables beneath each graph show groups that differed significantly by Mantel-Cox log-rank test. Significance set at 0.005 (Bonferroni correction for 5 groups). (B and E) Bacterial burdens (expressed as log10 CFU) over time (mean [SEM]). (C and F) AUC analysis. The median and 95% confidence interval are indicated for each group. Comparison across groups made by one-way ANOVA were significant (P < 0.0001; Kruskal-Wallis test). Pairwise comparisons between groups were performed using Dunn’s post hoc test. *P < 0.05; ****P < 0.0001. Data associated with this figure can be found in the supplemental data file (S1 Data). AUC, area under curve; C4BP, C4b-binding protein; CFU, colony-forming units; FH, factor H; mAb, monoclonal antibody; Tg, transgenic. (TIF) [file pbio.3000323.s007.TIF]

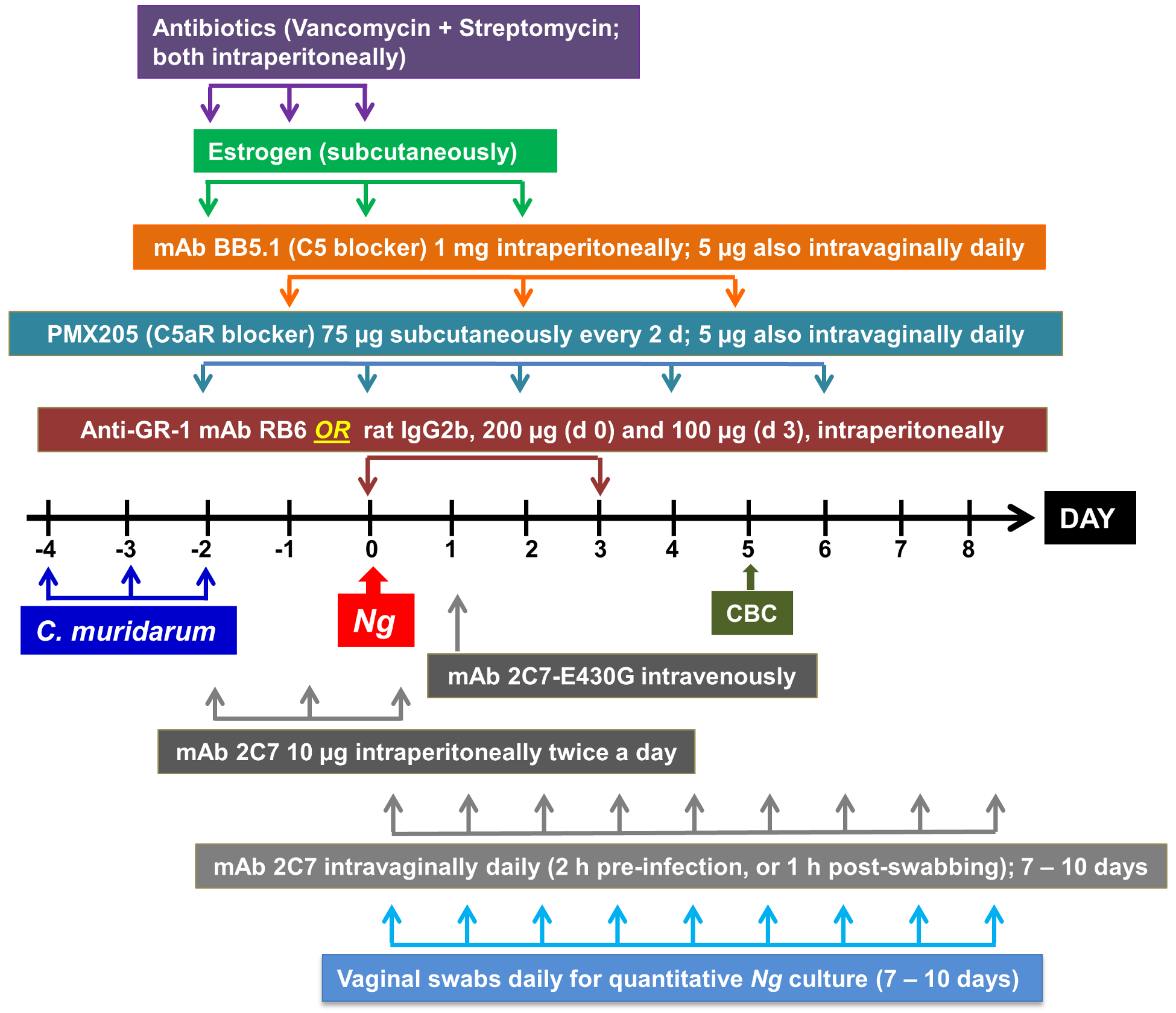

Supplement: S8 Fig — CBC, complete peripheral blood count; mAb, monoclonal antibody; Ng, N. gonorrhoeae; PMN, polymorphonuclear neutrophil. (TIF) [file pbio.3000323.s008.tif]
